# Development of an LC-MS/MS peptide mapping protocol for the NISTmAb

**DOI:** 10.1007/s00216-018-0848-6

**Published:** 2018-02-07

**Authors:** Trina Mouchahoir, John E. Schiel

**Affiliations:** 1 Biomolecular Measurement Division, National Institute of Standards and Technology, 100 Bureau Drive, Gaithersburg, MD 20899 USA; 2 Institute for Bioscience and Biotechnology Research, 9600 Gudelsky Drive, Rockville, MD 20850 USA

**Keywords:** Peptide mapping, NISTmAb, RM 8671, Tryptic digestion, Mass spectrometry, Optimization

## Abstract

Peptide mapping is a component of the analytical toolbox used within the biopharmaceutical industry to aid in the identity confirmation of a protein therapeutic and to monitor degradative events such as oxidation or deamidation. These methods offer the advantage of providing site-specific information regarding post-translational and chemical modifications that may arise during production, processing or storage. A number of such variations may also be induced by the sample preparation methods themselves which may confound the ability to accurately evaluate the true modification levels. One important focus when developing a peptide mapping method should therefore be the use of sample preparation conditions that will minimize the degree of artificial modifications induced. Unfortunately, the conditions that are amenable to effective reduction, alkylation and digestion are often the same conditions that promote unwanted modifications. Here we describe the optimization of a tryptic digestion protocol used for peptide mapping of the NISTmAb IgG1κ which addresses the challenge of balancing maximum digestion efficiency with minimum artificial modifications. The parameters on which we focused include buffer concentration, digestion time and temperature, as well as the source and type of trypsin (recombinant vs. pancreatic; bovine vs porcine) used. Using the optimized protocol we generated a peptide map of the NISTmAb which allowed us to confirm its identity at the level of primary structure.

**Electronic supplementary material:**

The online version of this article (10.1007/s00216-018-0848-6) contains supplementary material, which is available to authorized users.

**Graphical abstract:**
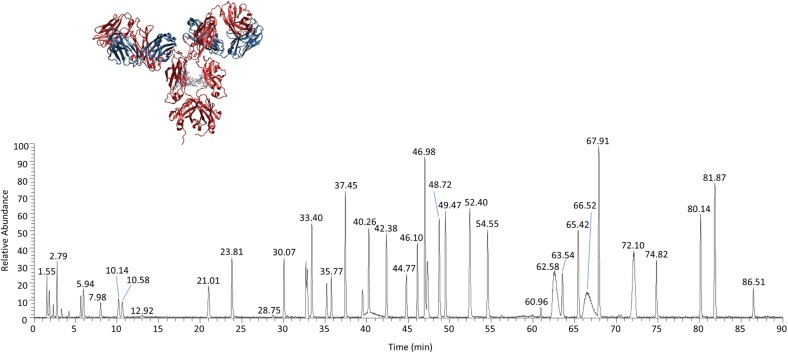
Peptide map of the NISTmAb RM 8671 monoclonal antibody. Tryptic digestion was performed using an optimized protocol and followed by LC-UV-MS analysis. The trace represents the total ion chromatogram. Each peak was mapped to peptides identified using mass spectrometry data.

## Introduction

Peptide mapping is a widely used technique for examining biopharmaceutical primary structure. Basic workflows employ bottom-up methodologies including enzymatic digestion followed by separation of the resulting peptides and analysis via ultraviolet (UV) detection and/or mass spectrometry (MS). The use of peptide mapping specifications as part of the suite of acceptance criteria used in the evaluation of biological products is outlined in Guideline Q6B published by the International Conference on Harmonization of Technical Requirements for Registration of Pharmaceuticals for Human Use (ICH) [1].

The ICH guidelines include the establishment of identity of drug substances and products via confirmation of the primary structure (i.e. amino acid sequence) as one of the major uses of peptide mapping [1]. In the quality control (QC) environment, identity is confirmed when the chromatographic profile of a peptide map conforms to expectation in comparison to a reference map (e.g. peak retention time, peak height, no new or missing peaks). Likewise, differences in the comparator peptide map are indicative of a change in, or degradation of, the drug substance/product. Thus, peptide mapping is also a valuable tool for evaluating the stability of reference standards. When coupled with mass spectrometry, changes in the landscape of the map can be pinpointed to a particular attribute, such as increased oxidation of a certain methionine residue [2–5] appearance of a new sequence variant [6–9] or changes in glycan composition [10–12]. These principles also apply to the use of peptide mapping techniques for the purposes of assessing biosimilarity to an originator drug product [13–19].

Peptide mapping can be used as an orthogonal tool to support primary structure analyses performed at the intact or protein subunit level and to provide additional site-specific information. For example, charge-based separations of intact proteins provide a birds-eye view of molecular status (i.e. global levels of deamidation), while peptide mapping techniques provide the ability to assign a specific location to the attribute. Because peptide mapping can provide a rather comprehensive and specific profile of a biological substance/product in one analytical package, efforts are being made to promote the development of qualified LC-MS peptide mapping assays for extended use in process monitoring and quality control [20]. Specificity is a key component of any analytical method used to evaluate the identity of a drug substance/product [21]. The peptide mapping method must therefore provide a high level of sequence coverage including the product-specific complementarity-determining regions (CDRs) to give the user confidence that no critical regions of the molecule go undetected. Optimization of a peptide map to minimize artificially induced variations that occur due to sample handling or processing provides confidence that changes in peak profiles are due to sample differences and are not artificially induced variations.

The three main “stages” in generating a peptide map are 1) enzymatic digestion, 2) peptide separation and 3) peptide detection. The most vulnerable of these stages to artificial modification is the process of producing peptides through enzymatic digestion. Here the sample may be exposed to various buffers, reducing and alkylating agents and even elevated temperatures. Many protein modifications, such as asparagine deamidation and methionine oxidation, are promoted by conditions such as elevated temperatures or high pH and are further exacerbated by exposure to these conditions for extended periods of time [22–32]. These factors should be considered when developing an optimal peptide mapping method geared toward minimizing artificial modifications.

Enzymatic digestion itself is prone to variation in regard to the efficiency and integrity of protein cleavage. The enzyme must reproducibly cleave the same locations to avoid introducing new peaks into or removing peaks from the chromatographic profile, whether due to missed cleavages, semi-tryptic cleavages or autolysis. Digestion conditions that promote the maximal efficiency of the enzyme are unfortunately also the same which can induce modification of the protein (e.g. incubation at 37 °C). Conversely, those conditions that may be optimal for denaturing and solubilizing the protein to thereby allow the enzyme easy access to cleavage sites are also conditions which will reduce the catalytic activity of the enzyme. Thus, finding optimal digestion conditions is not a clear cut process and often involves finding a balance between what is optimal for one component versus another.

Herein we describe optimization of a tryptic digestion method for use in the peptide mapping evaluation of the IgG1κ monoclonal antibody NISTmAb RM 8671 (NISTmAb) [33]. We took a step-wise approach to optimizing many parameters, with a specific focus on sample preparation/digestion. Our goal was to minimize exposure of the protein to those extremes that promote modification and variability. The optimized NISTmAb digest reported provides a common protocol for a sample preparation method that has historically varied significantly from lab to lab. Application of the digest to RM 8671 provides a framework that may prove useful in future comparisons of analytical technology performance within and between stakeholder labs and technologies. The optimized protocol was implemented with LC-MS/MS peptide mapping as the control strategy for NISTmAb primary amino acid sequence confirmation.

## Materials and methods

### Samples and materials

NISTmAb Primary Sample 8670 (PS 8670) is an in-house standard comprising a single production lot of NISTmAb [33]. PS 8670 and RM 8671 are both formulated in 12.5 mmol/L L-histidine/12.5 mmol/L L-histidine HCl, pH 6.0 at 10 mg/mL. Guanidine HCl (Cat #RDD001), Tris(hydroxymethyl)aminomethane (Cat #T6066), Tris(hydroxymethyl)aminomethane HCl (Cat #T5941), ethylenediaminetetraacetic acid (EDTA) (Cat #39692), urea (Cat #U0631), acetic acid (Cat #695084), iodoacetamide (IAM) (Cat #A3221), recombinant porcine trypsin expressed in *P. pastoris* (Cat #03708985001), trypsin purified from bovine pancreas (Cat #TRYPSEQM-RO) and trypsin purified from porcine pancreas (Cat #T6567) were purchased from Sigma Aldrich. Recombinant bovine trypsin expressed in corn (Cat #PRO-313) and recombinant human-2 trypsin expressed in *E. coli* (Cat # PRO-770) were purchased from ProSpec. Additional trypsin purified from porcine pancreas (Cat #V5280) was purchased from Promega. Dithiothreitol (DTT) (Cat #20291), Zeba™ Spin 7 K MWCO size-exclusion desalting columns (Cat #89882), LC/MS grade water (Cat # W6212), 0.1% formic acid in water (Cat # LS118) and 0.1% formic acid in acetonitrile (Cat #LS120) were purchased from Fisher Scientific. The C8 liquid chromatography column (AdvanceBio RP-mAb SB-C8, 2.1 mm ID × 150 mm, 3.5 um particle, 450 Å pore, Cat #783775–906) was purchased from Agilent Technologies and the C18 column (XSelect Peptide CSH C18 XP, 2.1 mm ID × 150 mm, 2.5 μm particle, 130 Å pore, Cat #186006727) was purchased from Waters Corp.

### Instrumentation

Liquid chromatography was performed using the Dionex UltiMate™ Rapid Separation Binary Pump (P/N HPG-3200RS), coupled to a thermostatted rapid separation well plate autosampler (P/N WPS-3000TRS), thermostatted column oven (P/N TCC-3000RS), and variable wavelength detector (P/N VWD-3400RS) manufactured by Thermo Scientific (Waltham, MA). Mass spectrometry analyses were performed using the LTQ Orbitrap Elite (for tryptic digests generated for time/temperature optimization) or the LTQ Orbitrap Discovery XL (for subunit analysis, digests generated for trypsin species optimization and for final PS 8670/RM 8671 peptide maps) with a heated electrospray ionization source probe (HESI-II) manufactured by Thermo Scientific, Waltham, MA. The instruments were controlled using Xcalibur 2.1.0 SP1 Build 1160 (Thermo Scientific, Waltham, MA) and Dionex Chromatography MS Link (DCMS Link) for Xcalibur 2.14 Build 3818 (Thermo Scientific, Waltham, MA).

### Sample preparation for peptide mapping (optimized tryptic digestion protocol)

The detailed buffer preparation and digestion protocol performed at scale can be found in the Electronic Supplementary Material (ESM) Document S1. In general, PS 8670 or RM 8671 was diluted to 1.0 mg/mL with denaturing buffer comprising 6 mol/L guanidine HCl, 1 mmol/L EDTA in 0.1 mol/L Tris(hydroxymethyl)aminomethane/Tris(hydroxymethyl)aminomethane HCl (Tris), pH 7.8. Reduction was achieved by the addition of 500 mmol/L dithiothreitol (DTT) to a final concentration of 5 mmol/L, followed by incubation at 4 °C for 60 min. Alkylation was performed by adding 500 mmol/L iodoacetamide (IAM) to a final concentration of 10 mmol/L and incubating at 4 °C for 60 min, in the dark. The denaturing buffer was exchanged to digestion buffer (1 mol/L urea in 0.1 mol/L Tris, pH 7.8) using Zeba™ Spin 7 K MWCO size-exclusion desalting columns (P/N 89882) (Thermo Scientific, Waltham, MA) according to the manufacturer’s instructions. Recombinant porcine trypsin (purchased from Sigma, Cat # 03708985001) was added at a 1:18 (enzyme:sample) mass ratio (based on NISTmAb protein concentration as measured by UV-Vis spectrophotometry after buffer exchange), the concentration of IgG was adjusted to 0.5 μg/μL and digestion allowed to proceed during a 4 h incubation at room temperature. When the digestion was complete, 0.1% formic acid in LC-MS grade water was added at a 1:1 volume ratio. Digests were stored at −80 °C until analysis.

For steps that required the addition of a small volume of concentrated stock solution to achieve a final, more dilute concentration in the working sample (e.g. “Reagent X was added to a final concentration of Y”), the volume of stock solution to be added was calculated using the following equation:

1 $$ {V}_1=\frac{V_{init}{M}_2}{M_1-{M}_2} $$

where M_1_ = stock solution concentration, M_2_ = desired final concentration, V_1_ = volume of stock solution to add, and V_init_ = volume of sample solution before addition of stock solution.

### LC-MS/MS analysis of tryptic digests

2.5 μg (10 μL) of peptide digests were loaded via autosampler onto a C18 column enclosed in a thermostatted column oven set to 40 °C. Samples were held at 7 °C while queued for injection. The chromatographic method was initiated with 98% Mobile Phase A (a 0.1% volume fraction of formic acid in water) and 2% Mobile Phase B (a 0.1% volume fraction of formic acid in acetonitrile) with the flow rate set at a constant 0.200 mL/min. After a 10 min wash, peptides were eluted over a 110 min gradient in which Mobile Phase B content rose at a rate of 0.39% per min to reach a final composition comprising 45% Mobile Phase B. Prior to the next sample injection, the column was washed for 15 min with 97% Mobile Phase B, then equilibrated at 98% Mobile Phase A for 25 min. The eluate was diverted to waste for the first 1.5 min and final 5 min of the run.

Peptides eluting from the chromatography column were analyzed by UV absorption at 214 nm followed by mass spectrometry on the LTQ Orbitrap Elite or Discovery XL. Replicate peptide mapping data were collected for PS 8670 and RM 8671 samples to include three tandem MS (MS/MS) analyses and one MS-only analysis each.

The MS/MS analyses were performed for peptide identification in data-dependent mode in which one cycle of experiments consisted of one full MS scan of 300 *m/z* to 2000 *m/z* followed by five sequential MS/MS events performed on the first through fifth most intense ions detected at a minimum threshold count of 500 in the MS scan initiating that cycle. The MS^n^ AGC target was set to 1E4 with microscans = 3. The ion trap was used in centroid mode at normal scan rate to analyze MS/MS fragments. Full MS scans were collected in profile mode using the high resolution FTMS analyzer (*R* = 30,000) with a full scan AGC target of 1E6 and microscans = 1.

Ions were selected for MS/MS using an isolation width of 2 Da, then fragmented by collision induced dissociation (CID) with helium gas using a normalized CID energy of 35, an activation Q of 0.25 and an activation time of 10 msec. A default charge state was set at *z* = 2. Data dependent masses were placed on the exclusion list for 45 s if the precursor ion triggered an event twice within 30 s; the exclusion mass width was set at ±1 Da. Charge state rejection was enabled for unassigned charge states. A rejection mass list included common contaminants at 122.08 *m/z*, 185.94 *m/z*, 355.00 *m/z*, 371.00 *m/z*, 391.00 *m/z*, 413.30 *m/z*, 803.10 *m/z*, 1222.10 *m/z*, 1322.10 *m/z*, 1422.10 *m/z*, 1522.10 *m/z*, 1622.10 *m/z*, 1722.10 *m/z*, 1822.10 *m/z*, and 1922.10 *m/z*.

MS-only analyses were performed for the generation of the TIC peptide map. These comprised full MS profile scans at *R* = 30,000 with a full scan AGC target of 1E6 and microscans = 1.

### Peptide mapping data analysis for optimized digests

The following data analyses were performed on optimized tryptic digest samples to map peptide identifications to TIC and UV peaks in the peptide map. *In-silico* peptide identification was performed on LC-MS/MS data using Byonic v 2.7.2 (Protein Metrics Inc., San Carlos, CA) [34]. Mass spectra were searched against the NISTmAb amino acid sequence, the human proteome (for identification of potential human contaminants), the mouse proteome (for identification of potential host cell protein contaminants) and the *Pichia pastoris* proteome (for identification of potential contaminants from the recombinant trypsin host cell). Protein sequences used to compile the database were obtained from UniProtKP (*www.uniprot.org*; downloaded 01–12–2016). Decoy sequences were also incorporated during the search.

Byonic search parameters were set to include peptides cleaved C-terminal to Arg and Lys residues, allowing for any number of missed cleavages as well as non-specific cleavage. Precursor mass tolerance was set to 10 ppm with fragment mass tolerance at 0.5 Da. The maximum precursor mass considered for analysis was 10,000 Da. Post-translational modifications considered within the search parameters included Carbamidomethylation (Cys, +57.021464 Da), Ammonia-loss/ Succinimide Formation (Asn; −17.026549 Da), Deamidation (Asn; +0.984016 Da), Dehydration (Asp, Ser, Thr, Tyr; −18.010565 Da), Carbamylation (Protein N-Terminus, Cys, Lys, Met, Arg, Ser, Thr, Tyr; +43.005814 Da), Dioxidation (Trp; +31.989829 Da), Gln - > pyro-Glu (N-terminal Gln; −17.026549 Da), Oxidation (Met; +15.994915 Da), Lys-loss (Protein C-Terminus; −128.094963 Da). All modifications were considered variable, with the exception of carbamidomethylation of Cys which was set as a fixed modification.

Glycopeptides were identified in the samples following a separate, more focused search using the 182 human N-glycan, no multiple fucose database and the parameters given above with the following exceptions: 1) only the NISTmAb sequence and decoy sequences were included in the database; 2) the maximum number of missed cleavages considered was two; 3) only fully specific cleavages were considered; 3) the only other modification considered was the fixed carbamidomethylation of Cys residues; 4) precursor and fragment ion masses were recalibrated using the Preview algorithm (Protein Metrics Inc., San Carlos, CA) v2.7.4 prior to search; 5) the fragment mass tolerance was 0.4 Da; and 6) parameters file was set to show all N-glycopeptides regardless of score or false discovery rate (FDR).

Manual analysis was used to verify the identification of glycopeptides and peptides ≤4 amino acid residues in length. Sequence coverage was calculated as a composition match at the peptide level, rather than a connectivity match at the amino acid level. In other words, individual amino acids were considered to be covered if their peptide of residence was identified, regardless of whether MS/MS fragmentation of the peptide produced full *y*- and *b*- ion series.

Peptide identifications were mapped to chromatographic peaks using ByoMap v 2.3 (Protein Metrics Inc., San Carlos, CA). The Xcalibur RAW file generated from the MS1 only analysis of the PS 8670 tryptic digest was used as the reference total ion chromatogram, while peptide identifications were imported from the Byonic search of MS/MS data using the following filters: 1) Byonic search score > 20; 2) a precursor *m/z* error < ± 10 ppm; and 3) minimum alternate rank score/primary rank score > 0.95. TIC peaks were picked as those having a minimum peak area of > 0.5 % of the  area of the sum of all peaks in the chromatogram. Peak boundaries and mapped peptide identifications were manually reviewed. Peptides identified from additional searches (e.g. targeted glycopeptide search) were manually incorporated into the peptide map.

The average retention time (RT), standard deviation and relative standard deviation (RSD) used to establish peak retention times for the PS 8670 reference map were calculated for corresponding peaks across chromatograms generated from four injections of the same PS 8670 tryptic digest.

### Tryptic digest optimization

PS 8670 was processed using individual method variations of what was ultimately found to be the optimal protocol. Peptide identification and extracted ion chromatogram (XIC) quantification of IgG peptides was performed on RAW data files generated during tryptic digest optimization using the NIST MSQC Pipeline (http://chemdata.nist.gov) [35, 36] in full mode with an in-house curated mass spectral library specific to the NISTmAb sequence (niggit2014725 library; obtained from Standard Reference Data Program, NIST, MML, Biomolecular Measurement Division). To identify trypsin autolysis products generated from the full array of trypsin species (i.e. porcine, bovine, human) LC-MS/MS data were submitted to the Byonic platform with a database comprising sequences for the trypsin species used (porcine trypsin UniProtKB ID #P00761; bovine trypsin UniProtKB ID #P00766, #Q7M3E1, #P00767, #P00760, and #Q29463; and human trypsin UniProtKB ID #P07478). XIC values were generated manually for trypsin autolysis peptides using the QualBrowser algorithm within Xcalibur using a mass tolerance of 10 ppm.

Various aspects of digestion efficiency and induced modifications were calculated as follows:

For optimization of urea concentration, digestion time and temperature, relative levels of non-specific cleavage were calculated as:

2 $$ \frac{\sum \mathrm{XICs}\ \mathrm{of}\ \mathrm{peptides}\ \mathrm{with}\ \mathrm{non}\hbox{-} \mathrm{specific}\ \mathrm{cleavage}}{\sum \mathrm{XICs}\ \mathrm{of}\ \mathrm{all}\ \mathrm{identified}\ \mathrm{peptides}}\times 100=\%\kern0.5em \mathrm{non}\hbox{-} \mathrm{specific}\ \mathrm{cleavage} $$

Peptides that were identified as non-specific cleavage but had the same retention time as a “parent” peptide with specific cleavage were considered as in-source fragments and thus were not counted as non-specifically cleaved peptides.

relative levels of missed cleavages were calculated as:

3 $$ \frac{\sum \mathrm{XICs}\ \mathrm{of}\ \mathrm{peptides}\ \mathrm{with}\ \mathrm{missed}\ \mathrm{cleavage}\mathrm{s}}{\sum \mathrm{XICs}\ \mathrm{of}\ \mathrm{all}\ \mathrm{identified}\ \mathrm{peptides}}\times 100=\%\kern0.5em \mathrm{missed}\ \mathrm{cleavage} $$

relative levels of trypsin autolysis were calculated as:

4 $$ \frac{\sum \mathrm{XICs}\ \mathrm{of}\ \mathrm{trypsin}\ \mathrm{peptides}}{\sum \mathrm{XICs}\ \mathrm{of}\ \mathrm{all}\ \mathrm{identified}\ \mathrm{peptides}}\times 100=\%\kern0.5em \mathrm{trypsin}\ \mathrm{autolysis} $$

relative levels of asparagine modification were calculated as:

5 $$ \frac{\sum \mathrm{XICs}\ \mathrm{of}\ \mathrm{peptides}\ \mathrm{with}\ \mathrm{deamidation}\ \mathrm{or}\ \mathrm{succinimide}\ \mathrm{formation}}{\sum \mathrm{XICs}\ \mathrm{of}\ \mathrm{all}\ \mathrm{identified}\ \mathrm{peptides}\ \mathrm{containing}\ \mathrm{Asn}}\times 100=\%\kern0.5em \mathrm{asparagine}\ \mathrm{modification} $$

relative levels of methionine oxidation were calculated as:

6 $$ \frac{\sum \mathrm{XICs}\ \mathrm{of}\ \mathrm{peptides}\ \mathrm{with}\ \mathrm{Met}\ \mathrm{oxidation}}{\sum \mathrm{XICs}\ \mathrm{of}\ \mathrm{all}\ \mathrm{identified}\ \mathrm{peptides}\ \mathrm{containing}\ \mathrm{Met}}\times 100=\%\kern0.5em \mathrm{oxidation} $$

total intensity of identified peptides was calculated as:

7 $$ \sum \mathrm{XICs}\kern0.62em \mathrm{of}\ \mathrm{all}\ \mathrm{identified}\ \mathrm{peptides}=\mathrm{total}\ \mathrm{intensity} $$

### Subunit analysis

5 μg of reduced or reduced/alkylated PS 8670 were loaded via autosampler onto a C8 column (AdvanceBio RP-mAb SB-C8, 2.1 mm ID × 150 mm, 3.5 um particle, 450 Å pore) (Agilent Technologies, Santa Clara, CA; P/N 783775–906) enclosed in a thermostatted column oven set to 40 °C. Samples were held at 7 °C while queued for injection. The chromatographic method was initiated with 90% Mobile Phase A (a 0.1% volume fraction of formic acid in water) and 10% Mobile Phase B (a 0.1% volume fraction of formic acid in acetonitrile) with the flow rate set at a constant 0.200 mL/min. After a 5 min wash, subunits were eluted over a 30 min gradient in which Mobile Phase B content rose at a rate of 1.67% per min to reach a final composition comprising 60% Mobile Phase B. Prior to the next sample injection, the column was washed for 5 min with 95% Mobile Phase B, then equilibrated to 10% Mobile Phase A for 5 min. The eluate was diverted to waste for the first 5 min and final 1 min of the run.

Heavy and light chain species eluting from the chromatography column first passed through a variable wavelength detector (Dionex UltiMate™ 3000 Variable Wavelength Detector) (Thermo Scientific, Waltham, MA; P/N VWD-3400RS) set to measure UV absorption at 280 nm. Ions were then introduced into an LTQ Orbitrap Discovery mass spectrometer fitted with a heated electrospray ionization source probe (HESI-II). MS data were collected in the 300 *m/z* to 2000 *m/z* range with a resolving power of 30,000.

### Deconvolution

Deconvolution of MS data collected from PS 8670 subunits was performed using the Manual ReSpect™ algorithm found in Protein Deconvolution v 4.0 (Thermo Scientific, Waltham, MA). Theoretical masses were calculated using the NIST Mass and Fragment Calculator v1.3 [37] and the NIST defined elemental average masses.

## Results and discussion

### Digest optimization

Many artificial modifications induced during sample preparation are dependent on temperature, duration of incubation or reaction with the digestion reagents themselves [22–25, 29, 38]. We therefore evaluated variations in reaction length, temperature and buffer composition.

Taking a step-wise approach we first examined the denaturing conditions under which the protein is prepared for tryptic digestion. For this and all optimization studies described we used the NISTmAb Primary Sample 8670 (PS 8670) which is an in-house standard comprising a single production lot of NISTmAb.

#### Optimization of denaturing reagent

Our previous platform method called for the use of a buffer comprising 6 mol/L guanidine HCl, 1 mmol/L EDTA in 0.1 mol/L Tris, pH 7.8, to aid in the denaturing of the protein prior to tryptic digestion. It is important that the protein is sufficiently denatured, unfolded, and reduced prior to digestion in order that the enzyme has access to every region of the protein and can thereby accomplish a complete digestion. Although this buffer composition may be fully effective in denaturing the IgG, the presence of guanidine in the buffer poses a problem for the digestion itself since it will also denature trypsin. This necessitates the removal of the guanidine prior to digestion, a step that often causes loss of sample.

We sought to determine whether we could replace the guanidine with a buffer that could also be used for the digestion. We performed denaturation and reduction of PS 8670 by first diluting the antibody to 1.0 mg/mL with buffer comprising 1) 0.10 mol/L Tris, pH 7.8; B) 2.0 mol/L urea in 0.10 mol/L Tris, pH 7.8; or C) 6.0 mol/L guanidine HCl, 1 mmol/L EDTA in 0.10 mol/L Tris, pH 7.8. DTT was then added to a final concentration of 20 mmol/L and the samples incubated at 37 °C for one hour. We analyzed the samples via LC-UV-MS to determine levels of reduction into heavy and light chains. This also acted as a surrogate for measuring actual denaturation (i.e. protein unfolding) with the assumption that the greater degree to which the sample unfolds under each buffering condition, the greater access the DTT will have to its disulfide bonds. Thus, complete reduction of the protein is reflective of sufficient unfolding.

**Fig. 1:**
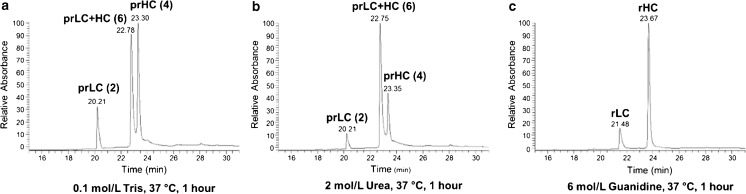
LC-UV chromatograms of denatured and reduced IgG. PS 8670 was denatured using (**a**) 0.10 mol/L Tris; (**b**) 2.0 mol/L urea; or (**c**) 6.0 mol/L guanidine HCl. Following incubation in 20 mmol/L DTT at 37 °C for 1 h, 5 μg of sample were analyzed by UV-LC-MS. The species comprising each UV peak were identified by deconvolution of their corresponding MS spectra. LC = light chain; HC = heavy chain; pr = partially reduced; r = reduced; the number of intact disulfide bonds is given in parentheses. Representative deconvoluted masses calculated for each species are listed in ESM Table S1

As shown in Fig. 1a and b, three major UV peaks were observed for samples denatured and reduced in either Tris alone or urea (representative observed masses are listed ESM Table S1). Deconvolution of the MS data collected for these three UV peaks showed that the species eluting at ≈ 20.2 min and ≈ 23.3 min contained free light and heavy chains, respectively, but that they were not completely reduced (i.e. the disulfide linkages between heavy and light chain were broken, but some intra-chain disulfides remained intact) (ESM Table S1). The peak at ≈ 22.7 min was found to contain heavy chain/light chain pairs indicating a lack of reduction of inter-chain disulfides. The sample that was denatured and reduced in the guanidine buffer produced two UV peaks with retention times distinct from those arising after reduction in the Tris and urea buffers (Fig. 1c). The deconvoluted masses determined for the species comprising the 21.48 min and 23.67 min peaks matched the theoretical masses of completely reduced light and heavy chains, respectively (ESM Table S1). Thus, only the guanidine buffer was effective in the complete denaturation and reduction of PS 8670 and is a necessary component of the digestion method.

Reducing the concentration of the chaotrope in the denaturing buffer by half, to 3.0 mol/L guanidine HCl, and keeping all other conditions the same resulted in the incomplete reduction of intrachain disulfides within both heavy and light chains (data not shown). Thus, we chose to continue using the denaturing buffer comprising 6 mol/L guanidine HCL (in 0.1 mol/L Tris, 1 mmol/L EDTA) to ensure complete disulfide bond reduction. It should be noted that 6 mol/L refers to the concentration of guanidine HCl in the prepared stock solution. In our protocol, after the addition of the IgG and the DTT solution to the denaturing buffer the actual concentration of guanidine HCl during the reduction step is 5.34 mol/L (see ESM Document S1).

#### Optimization of denaturation/reduction temperature

To determine whether we could denature and reduce the protein without the use of elevated temperatures which are a known proponent of protein modification [22, 23, 29], we diluted PS 8670 to 1 μg/μL with 6 mol/L guanidine HCl, 1 mmol/L EDTA in 0.1 mol/L Tris, pH 7.8, added DTT to a final concentration of 20 mmol/L and allowed reduction to proceed for 1 h at either 4 °C, room temperature (≈ 25 °C) or 37 °C. We analyzed the samples via LC-UV-MS to determine whether the samples were fully reduced under each condition. All samples were fully reduced to heavy and light chains regardless of incubation temperature (ESM Fig. S1), suggesting that there is no need to expose the protein to elevated temperatures (i.e. 37 °C, or even room temperature) for complete reduction to take place.

#### Optimization of reducing reagent concentration

We chose to work with dithiothreitol (DTT) rather than tris(2-carboxyethyl)phospine (TCEP) as our reducing agent because TCEP can reduce oxidized methionine residues [39] present on the molecule prior to digestion which could result in inaccurate quantitation of that attribute. The presence of DTT in the buffer can also be an issue since it reportedly promotes methionine oxidation following the metal-catalyzed reduction of oxygen to hydrogen peroxide [40–43]. However, we strove to circumvent this by including EDTA in the denaturing buffer [39] as well as keeping DTT concentrations low during protein reduction. Our previous platform called for disulfide bond reduction using a DTT concentration of 20 mmol/L. We tested the efficacy of reducing the IgG at 10 mmol/L DTT and 5 mmol/L DTT in the 6 mol/L guanidine denaturing buffer at 4 °C for one hour and using LC-UV-MS found that a DTT concentration as low as 5 mmol/L indeed provided reduction into heavy and light chains with complete reduction of intrachain disulfides (ESM Fig. S2). We approximate that the conditions we used provided a 20- to 25- fold molar excess of DTT over cysteine and note that it is important to maintain a ratio of 5 mmol DTT per 1 μg/μL of NISTmAb for complete reduction. If the IgG is reduced at a higher concentration, the concentration of DTT must be increased proportionally (data not shown).

#### Optimization of reducing time

**Fig. 2:**
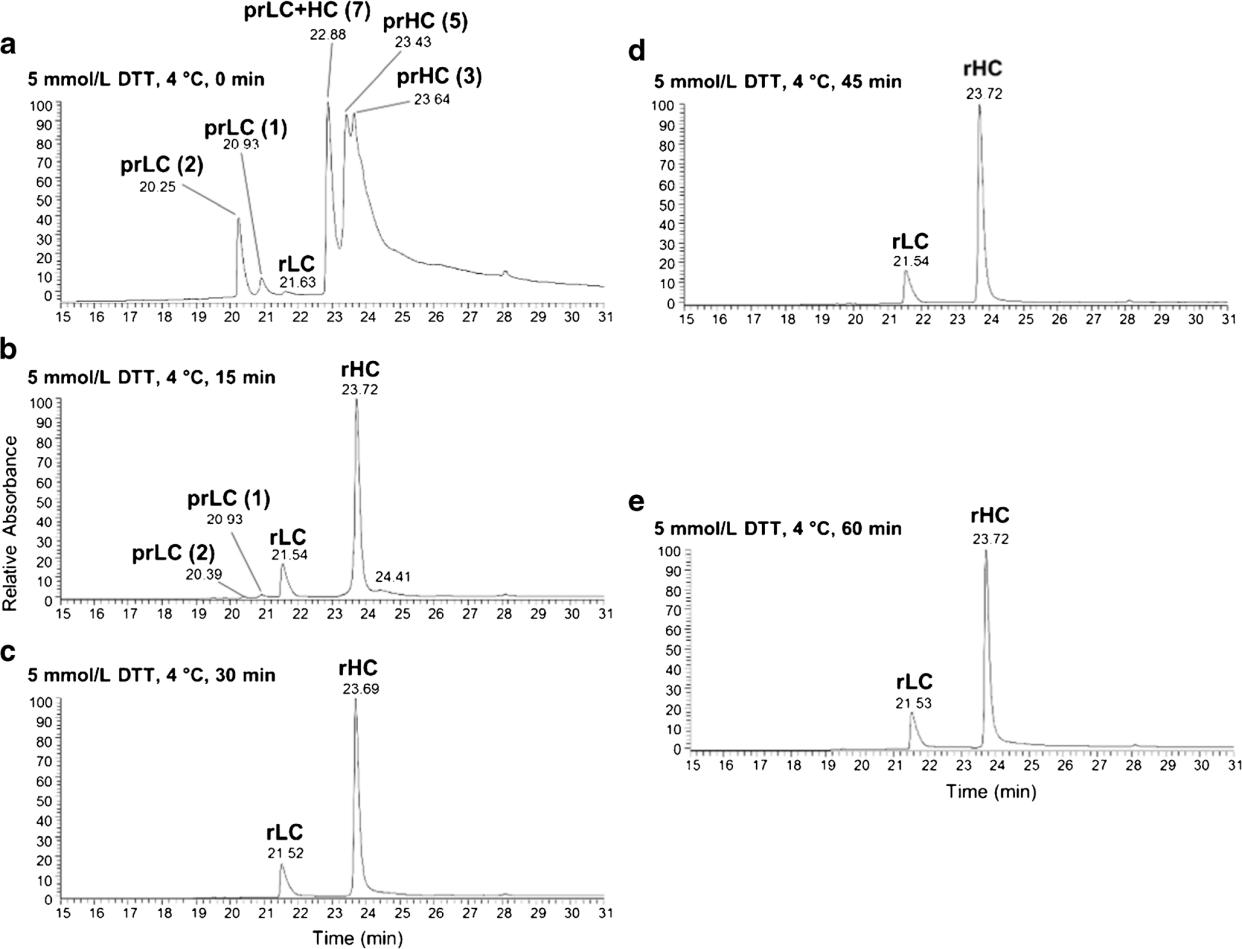
LC-UV chromatograms of IgG reduced for varying lengths of time. PS 8670 was reduced at 4 °C in 6.0 mol/L guanidine HCl buffer with 5 mmol/L DTT for (**a**) 0 min, (**b**) 15 min, (**c**) 30 min, (**d**) 45 min, or (**e**) 60 min. The level of reduction was determined by LC-UV-MS analysis of 5 μg of mAb. The species comprising each UV peak were identified by deconvolution of their corresponding MS spectra. LC = light chain; HC = heavy chain; pr = partially reduced; r = reduced; the number of intact disulfide bonds is given in parentheses. Representative deconvoluted masses calculated for each species are listed in ESM Table S1

Finally, we examined incubation times needed to fully reduce the mAb. The LC-UV-MS data in Fig. 2 shows that full reduction into heavy and light chains can be achieved using 5 mmol/L DTT in 6 mol/L guanidine denaturing buffer at 4 °C in as few as 30 min. In the event that some unreduced mAb remained below our level of detection we have chosen to extend this time to 60 min in our optimized method to ensure complete reduction.

#### Optimization of alkylation conditions

**Fig. 3:**
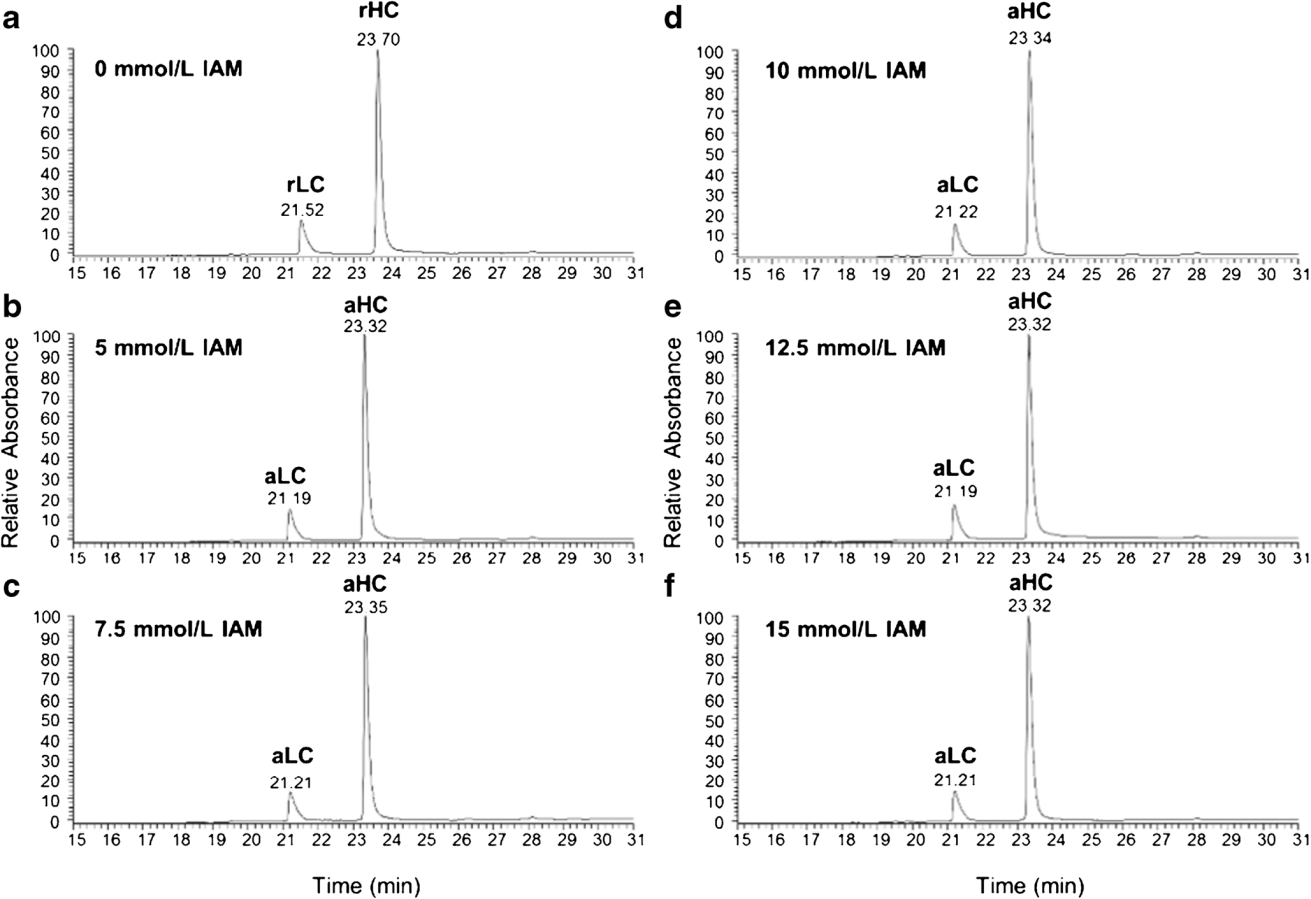
LC-UV chromatograms of IgG alkylated with varying IAM concentrations. PS 8670 was reduced with 5 mmol/L DTT at 4 °C for 1 h, followed by alkylation with (**a**) 0 mmol/L; (**b**) 5 mmol/L; (**c**) 7.5 mmol/L; (**d**) 10 mmol/L; (**e**) 12.5 mmol/L; or (**f**) 15 mmol/L iodoacetamide (IAM). The level of alkylation was determined by LC-UV-MS analysis of 5 μg of mAb. The species comprising each UV peak were identified by deconvolution of their corresponding MS spectra. LC = light chain; HC = heavy chain; r = reduced; a = alkylated. Representative deconvoluted masses calculated for each species are listed in ESM Table S1

Following reduction, cysteine bonds must be alkylated to prevent reformation of the disulfide bridges. This is often achieved using iodoacetamide (IAM) or iodoacetic acid (IAA). We have traditionally used IAM due to its faster reaction time [44, 45], and because unlike IAA it does not introduce a negative charge to the derivatized peptide. Since any remaining DTT competes with the sulfhydryl group for the alkylating agent, IAM is typically used in excess of the reducing agent to ensure complete alkylation. However, using too high a concentration runs the risk of overalkylation of the protein, including alkylation on residues other than cysteine [46, 47]. We tested the use of increasing IAM concentrations (0 mmol/L, 5 mmol/L, 7.5 mmol/L, 10 mmol/L, 12.5 mmol/L and 15 mmol/L) to determine the lowest level necessary for complete cysteine alkylation of PS 8670. We noted earlier elution times for heavy and light chain peaks arising from all IAM-treated samples (Fig. 3b to e) compared to the sample with no alkylating agent (Fig. 3a). Complete alkylation was confirmed by deconvolution of the MS data corresponding to each UV peak (ESM Table S1). Our results indicate that alkylation in 6 mol/L guanidine denaturing buffer at 4 °C for one hour was complete even at an IAM concentration equal to that of the reducing agent (5 mmol/L) (Fig. 3b). No evidence of overalkylation was observed even at the highest concentration (15 mmol/L) tested. In the event that there remains some unalkylated/overalkylated sample below our level of detection (as peptide mapping will be more sensitive than subunit analysis) we have chosen to use a medial concentration of 10 mmol/L for our optimized method.

#### Optimization of tryptic digestion conditions

Following reduction and alkylation, many standard tryptic digests for monoclonal antibodies call for buffer exchange into a urea-containing digestion buffer to maintain solubility of the protein substrate. This, however, can also hinder digestion efficiency since the presence of urea slows the enzymatic activity of trypsin. Further, buffer composition, pH and temperature are also known to induce chemical modifications such as methionine oxidation and asparagine deamidation [22–32]. In order that we may find the appropriate balance between maintaining antibody solubility while promoting efficient trypsin activity and avoiding artificial modifications, we digested PS 8670 using either no urea (0.1 mol/L Tris, pH 7.8 only), 1.0 mol/L urea (in 0.1 mol/L Tris, pH 7.8), or 2.0 mol/L urea (in 0.1 mol/L Tris, pH 7.8). For each urea concentration we allowed the digestion to proceed for varied lengths of time and temperature: 1 h at either room temperature (≈ 25 °C) or 37 °C, or for 4 h at either 4 °C, room temperature or 37 °C.

**Fig. 4:**
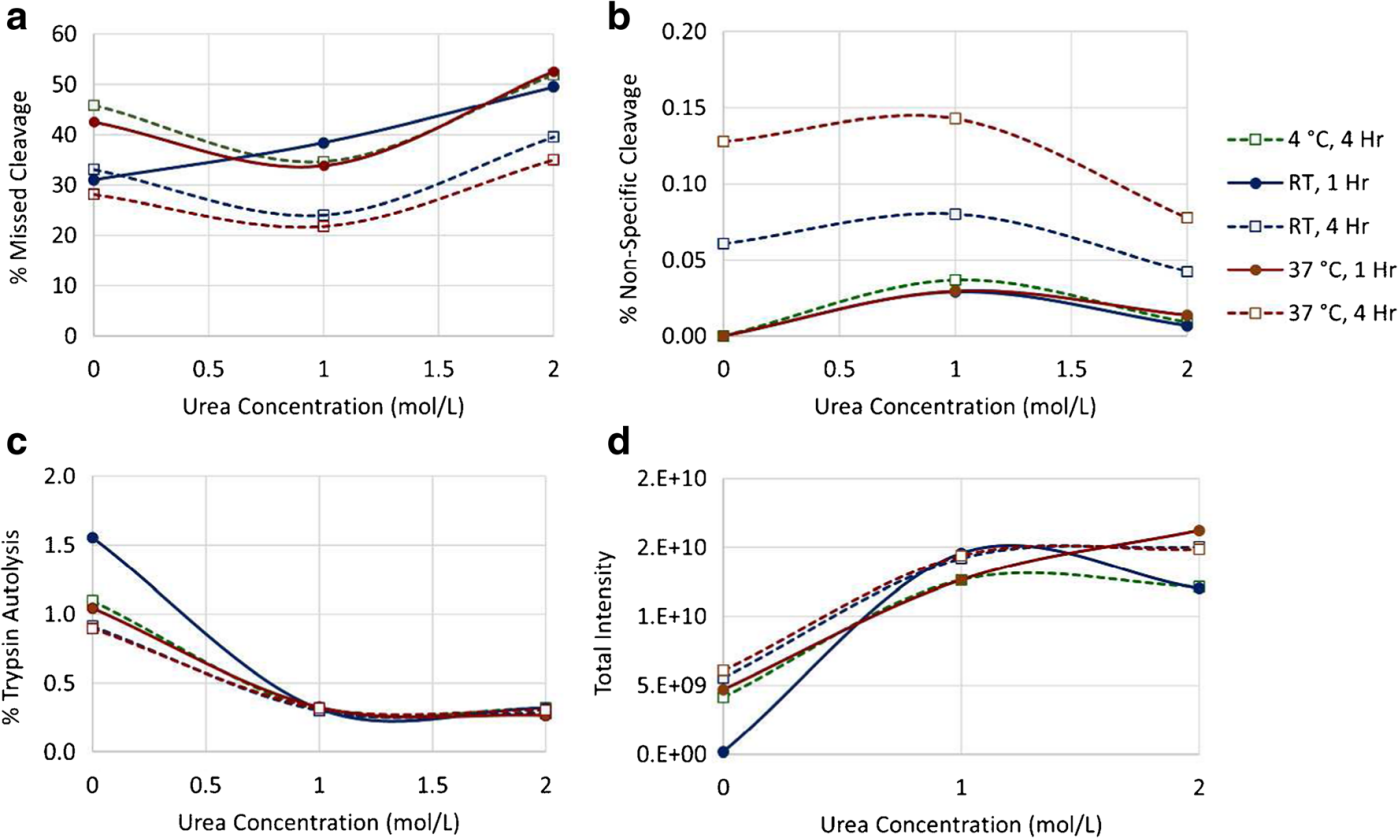
Relative digestion efficiency under varied conditions. PS 8670 was digested with trypsin under varied urea concentrations, digestion times and incubation temperatures followed by LC-MS/MS analysis. Digestion efficiency was evaluated by examining relative levels of (**a**) missed cleavage; (**b**) non-specific cleavage; (**c**) trypsin autolysis; and (**d**) total intensity of identified peptides. RT = room temperature

Following LC-MS/MS analysis, we applied the MSQC algorithm for peptide identification and quantification and used these data to evaluate the quality of the digested samples. We first focused on the efficiency of the digestions themselves using relative levels of missed cleavage, non-specific cleavage (enzymatic cleavage C-terminal to a residue other than Lys or Arg) and trypsin autolysis as a set of metrics for this property (Fig. 4). Each of these are best kept at a minimum because they may unnecessarily complicate the peptide map by increasing the number of peaks in the chromatogram. Evaluation of the data was done by comparing the trends observed as the urea concentration increased for samples digested under the same temperature and time conditions (a sample “set”).

Within each digestion set the lowest relative levels of missed cleavage were typically observed for the sample digested in 1.0 mol/L urea (Fig. 4a), indicating that this concentration provides sufficient IgG solubility to allow the enzyme to access the protein for digestion, but is not so high as to detrimentally inhibit digestion via denaturation of the enzyme. Not surprisingly, sample sets allowed to digest for the longer 4 h period and at ambient to physiological temperature exhibited overall lowest missed cleavages. The trends observed for missed cleavages were reversed for observed levels of non-specific cleavage, with each sample set having its highest levels rising from the 1.0 mol/L urea digest (Fig. 4b). Non-specific cleavage also increased with time and temperature when comparing sample sets.

Finally, we compared trypsin autolysis levels across the samples sets and saw that all samples digested in 1.0 mol/L urea and 2.0 mol/L urea regardless of time or temperature conditions had nearly the same low autolysis levels (Fig. 4c). Higher levels were observed for all times and temperatures when samples were digested without urea. It is possible that this is due to a lack of IgG solubility in the absence of a chaotropic agent, which effectively lowers the concentration of IgG available for digestion and increases the interaction of trypsin with itself. This is supported by the trend observed when comparing the total intensities of identified PS 8670 peptides for each digest (Fig. 4d). Here total intensity was lowest for all samples digested without urea as compared to those digested with 1.0 mol/L urea or 2.0 mol/L urea, which had total intensities similar to each other at all time and temperature conditions. These data further negated consideration of excluding urea during digestion.

**Fig. 5:**
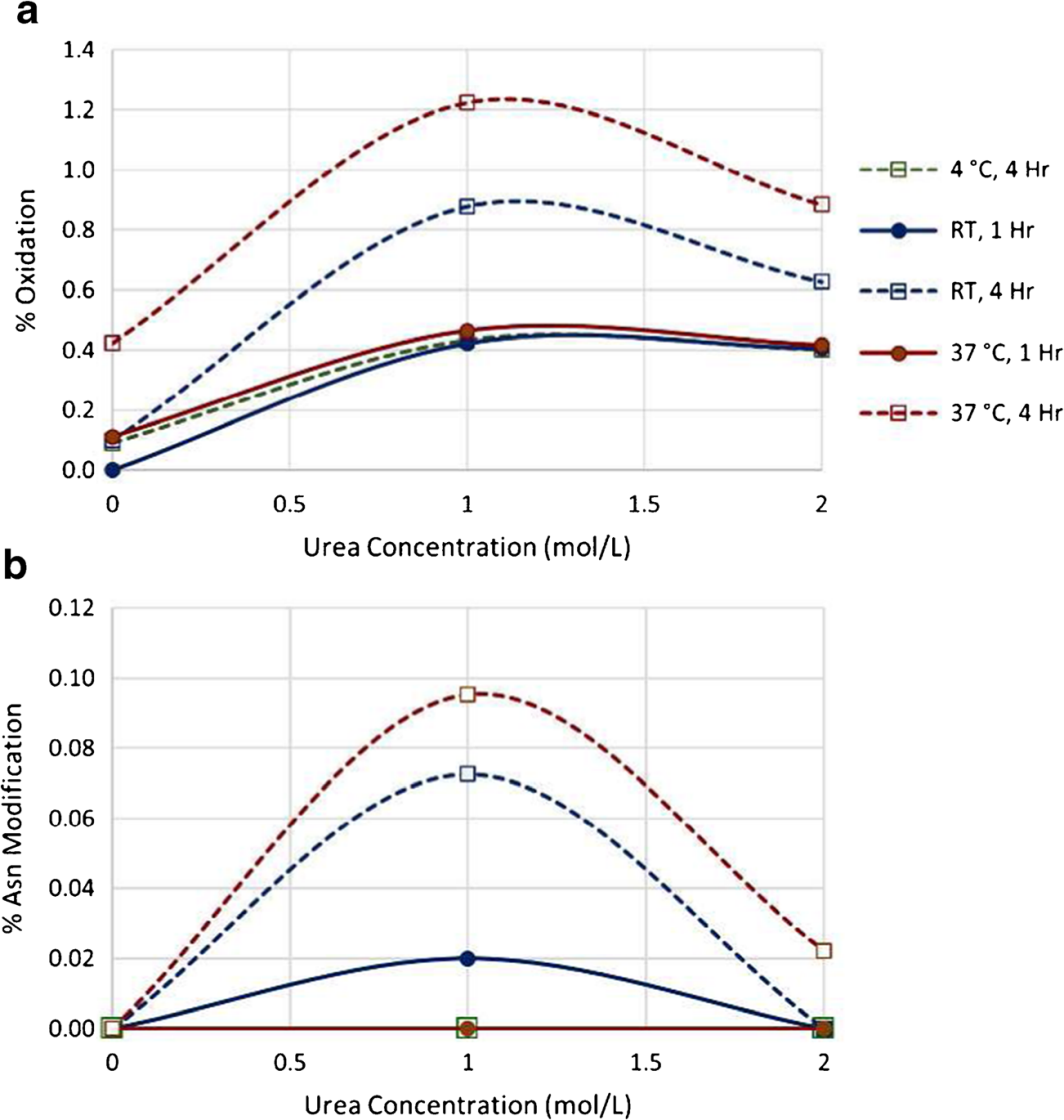
Relative levels of amino acid modification induced under varied digestion conditions. PS 8670 was digested with trypsin under varied urea concentrations, digestion times and incubation temperatures followed by LC-MS/MS analysis. Data were examined to determine relative levels of induced a) methionine oxidation and b) asparagine modification. RT = room temperature

Post-translational modifications are a main focus when evaluating the integrity of a biopharmaceutical molecule. Changes in attributes such as methionine oxidation or asparagine deamidation can be detrimental to the efficacy, stability and immunogenicity of a mAb and must therefore be closely monitored. Because some of these modifications may also be induced by the digestion process itself, it is important that a peptide mapping protocol minimize any artifacts that may confound the analysis of these attributes. The varied levels of methionine oxidation in our data indicated that some amount of modification could be attributed to the digestion method itself (Fig. 5). Samples digested for four hours had higher oxidation levels than their time/temperature counterparts digested for only one hour. Furthermore, samples digested for the same time period and same urea concentration saw an increase in oxidation as the temperature increased, with the highest levels observed for the sample digested in 1.0 mol/L urea at 37 °C for four hours (Fig. 5a). We observed this same trend with regard to asparagine modification, where levels increased as incubation times lengthened and temperatures were elevated within each sample set (Fig. 5b). Relative asparagine modification levels were highest, although <0.10%, for samples digested in 1.0 mol/L urea at 37 °C for four hours. No carbamylated species were detected in these analyses.

We reviewed the data collected from our various digestion conditions to make a determination as to which set of conditions were the most optimal to use going forward. The same time, temperature and buffer composition conditions that produced optimal results for one digestion parameter in some cases were the same conditions that led to the least optimal results for another parameter. Therefore, we sought out the set of conditions that appeared to offer the most reasonable balance between the observed extremes.

As a whole, it seemed that digestion performed in the absence of urea was sub-par in many regards. First and foremost, the low total intensity of detected peptides in non-urea samples indicates low solubility of these samples and likely larger amounts of undigested IgG in these samples as compared to those digested in urea (Fig. 4d). Secondly, non-urea containing samples had the highest levels of trypsin autolysis compared to all samples digested with urea (Fig. 4c). The 0 mol/L urea samples also competed with those digested in 2.0 mol/L urea for the highest levels of missed cleavages within each sample set (Fig. 4a). Although samples digested without urea had the lowest oxidation and asparagine modification levels within each set (Fig. 5), this does not outweigh the more negative factors listed here for these samples. Thus, we categorically rejected the possibility of performing the digestion without urea under any time or temperature conditions.

Samples digested in 2.0 mol/L urea had similar levels of total peptide intensity and trypsin autolysis as all samples digested in 1.0 mol/L urea (Fig. 4c, d). The 2.0 mol/L urea digests trended toward lower levels of non-specific cleavage, methionine oxidation and asparagine modification as compared to their time/temperature counterparts digested in 1.0 mol/L urea (Fig. 4b, Fig. 5). However, these improvements were modest (< 0.5 percentage point) compared to the increase in missed cleavage levels in 2.0 mol/L urea (> 11 percentage points, Fig. 4a). It was therefore determined that the 1.0 mol/L urea was the more optimal choice for tryptic digestion.

Finally, we evaluated which time and temperature conditions were optimal for digestion when performed in 1.0 mol/L urea. Missed cleavage levels were increased in all one hour digests as well as the four hour digest performed at 4 °C, thus these conditions were not considered further. Digestion for four hours at room temperature showed slightly higher missed cleavage rates, but lower non-specific cleavage and induced chemical modifications (Fig. 5) versus the 37 °C digestion. As a compromise between the numerous factors, we selected digestion in 1 mol/L urea at room temperature for 4 h as our optimal digest conditions.

#### Trypsin species and source

To this point we had used a trypsin enzyme purified from porcine pancreas for our digestions. We were interested to see whether digestion efficiency would be affected using trypsin from other species or sources. Therefore, we digested PS 8670 using either porcine, bovine or human species trypsin that had either been purified from pancreatic tissue or produced recombinantly. In addition, because some manufacturers recommend the addition of calcium to the digestion buffer to promote trypsin activity, we performed each digest with and without CaCl_2_.

**Fig. 6:**
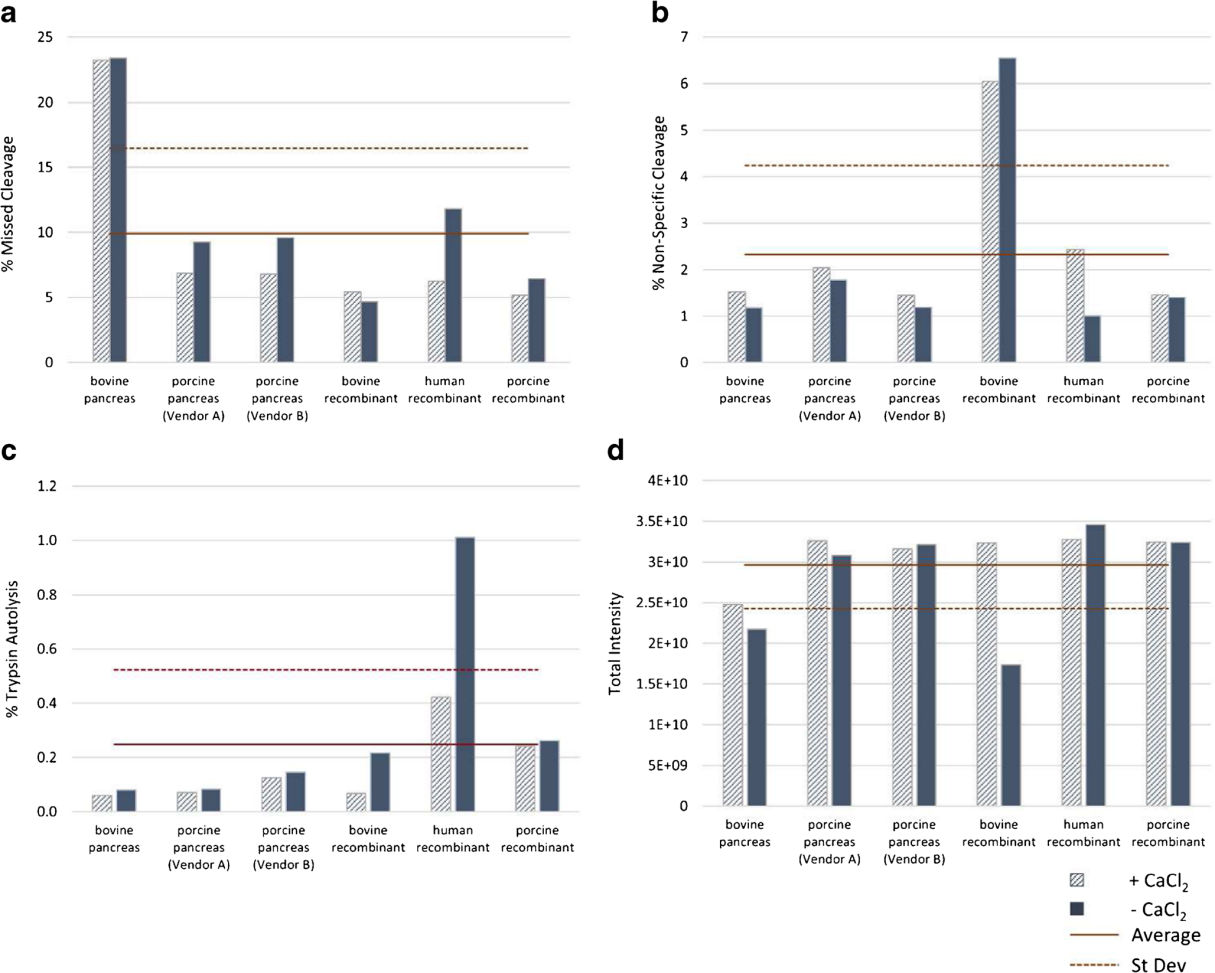
Relative digestion efficiency using different species and sources of trypsin. PS 8670 was digested with trypsin from different species (bovine, porcine or human) and sources (pancreatic or recombinant) followed by LC-MS/MS analysis. Digestion efficiency was evaluated by examining relative levels of (**a**) missed cleavage; (**b**) non-specific cleavage; (**c**) trypsin autolysis; and (**d**) total intensity of identified peptides

We analyzed each tryptic digest by LC-MS/MS followed by peptide identification and quantitation. Again we used relative levels of missed cleavage, non-specific cleavage, trypsin autolysis and total intensity to evaluate digestion efficiency of the samples. Although here we calculated the average value across the samples for each given parameter and considered those that fell outside one standard deviation of the average as sub-optimal (Fig. 6). This removed both pancreatic and recombinant bovine trypsin from consideration for use in future digests. The relative levels of missed cleavage observed in the digests using pancreatic bovine trypsin, with and without CaCl_2_, were well above one standard deviation of the average (Fig. 6a), and the samples digested with recombinant bovine trypsin resulted in non-specific cleavage levels that were also one standard deviation above average (Fig. 6b). The digests generated using pancreatic bovine trypsin also had total peptide intensity levels at or below one standard deviation of the average (Fig. 6d). Trypsin autolysis levels also fell outside our criteria for the sample digested using recombinant human trypsin when calcium was not included in the digestion buffer and were well above average levels when calcium was used. Therefore this trypsin type was also removed from further consideration (Fig. 6c).

The samples that remained in this analysis were those digested using porcine pancreas trypsin (two different vendors) and recombinant porcine trypsin. Among these candidates we did not see an appreciable difference between total peptide intensity values or non-specific cleavage (Fig. 6a, d). While trypsin autolysis was highest in the recombinant porcine digest, it was by a very small margin (< 0.2 percentage points) (Fig. 6c). Finally, the recombinant porcine digest was lowest in missed cleavages compared to the other two remaining candidates by 3 percentage points (Fig. 6a). Of the candidates remaining for consideration there was no one candidate that stood out as the obvious choice as an optimal trypsin source. We decided to move forward using the porcine recombinant trypsin for our peptide mapping digests on the assumption that a recombinant source may perform more consistently than one purified from pancreatic tissue.

Evaluation of the effect of CaCl_2_ on digestion efficiency showed a trend toward higher levels of missed cleavage (Fig. 6a) and trypsin autolysis (Fig. 6c) in the absence of calcium, but lower levels of non-specific cleavage under these conditions (Fig. 6b). The degree to which the presence of calcium affected these digestion parameters varied with each trypsin type. The advantage of including CaCl_2_ in the digestion buffer when using the porcine recombinant trypsin chosen as the final candidate seemed to be only slight in regard to missed cleavage and trypsin autolysis, and offered no improvement in specific cleavage or total intensity. Without seeing a strong benefit of using calcium during digestion we opted not to include it in our optimized protocol.

### Peptide mapping

#### Primary sample 8670 peptide map

**Fig. 7:**
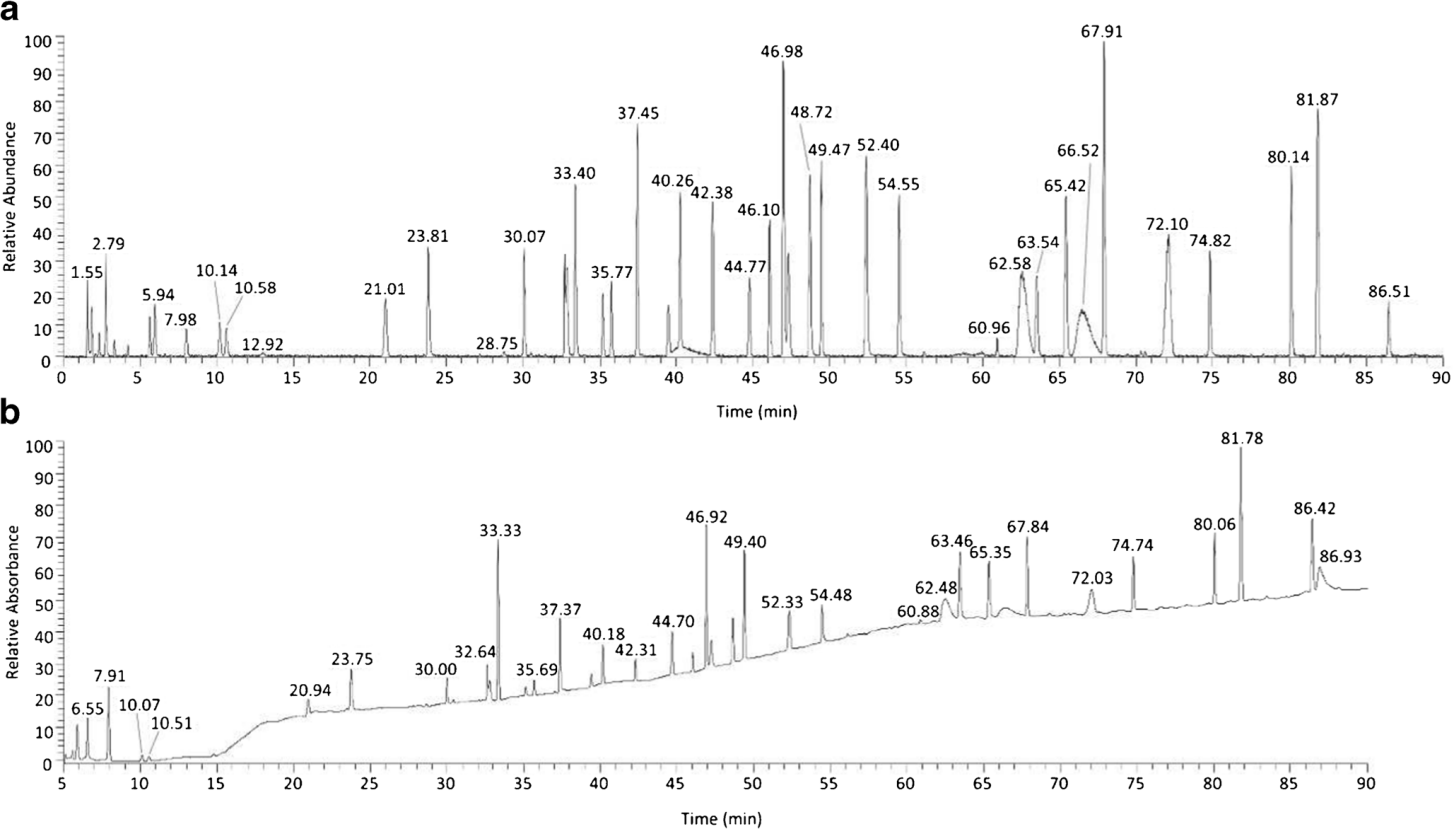
PS 8670 peptide map. The PS 8670 tryptic digest was analyzed by LC-UV-MS in quadruplicate. Mean retention times for TIC and UV chromatographic peaks were calculated and listed in ESM Table S2. These values are used here to label their corresponding peaks (panel a = TIC; panel b = UV 214 nm) and together constitute the peptide map. Zoomed views of the TIC and UV traces with all peaks labeled are found in ESM Fig. S3. The initial five minutes of the UV traces are not shown in panel b due to the large difference in scale between the relative levels of absorbance of peaks detected during the 0 min to 5 min period and the 5 min to 90 min period. This time period is depicted in ESM Fig. S3, panel b. A complete list of peak retention times and corresponding peptide identifications is given in ESM Table S2

We applied our optimized tryptic digest protocol (See ESM Document S1) to generate a peptide map of PS 8670. We analyzed the digest using LC-UV-MS to produce a TIC map comprising 54 peaks and a UV map comprising 56 peaks. Raw mass spectrometry data from LC-MS/MS analysis were subjected to interrogation by the Byonic algorithm for peptide identification. The ByoMap algorithm was used to match peptide identifications with TIC peaks having a minimum peak area > 0.5% of the area of the sum of all peaks (Fig. 7, ESM Fig. S3 and ESM Table S2).

**Fig. 8:**
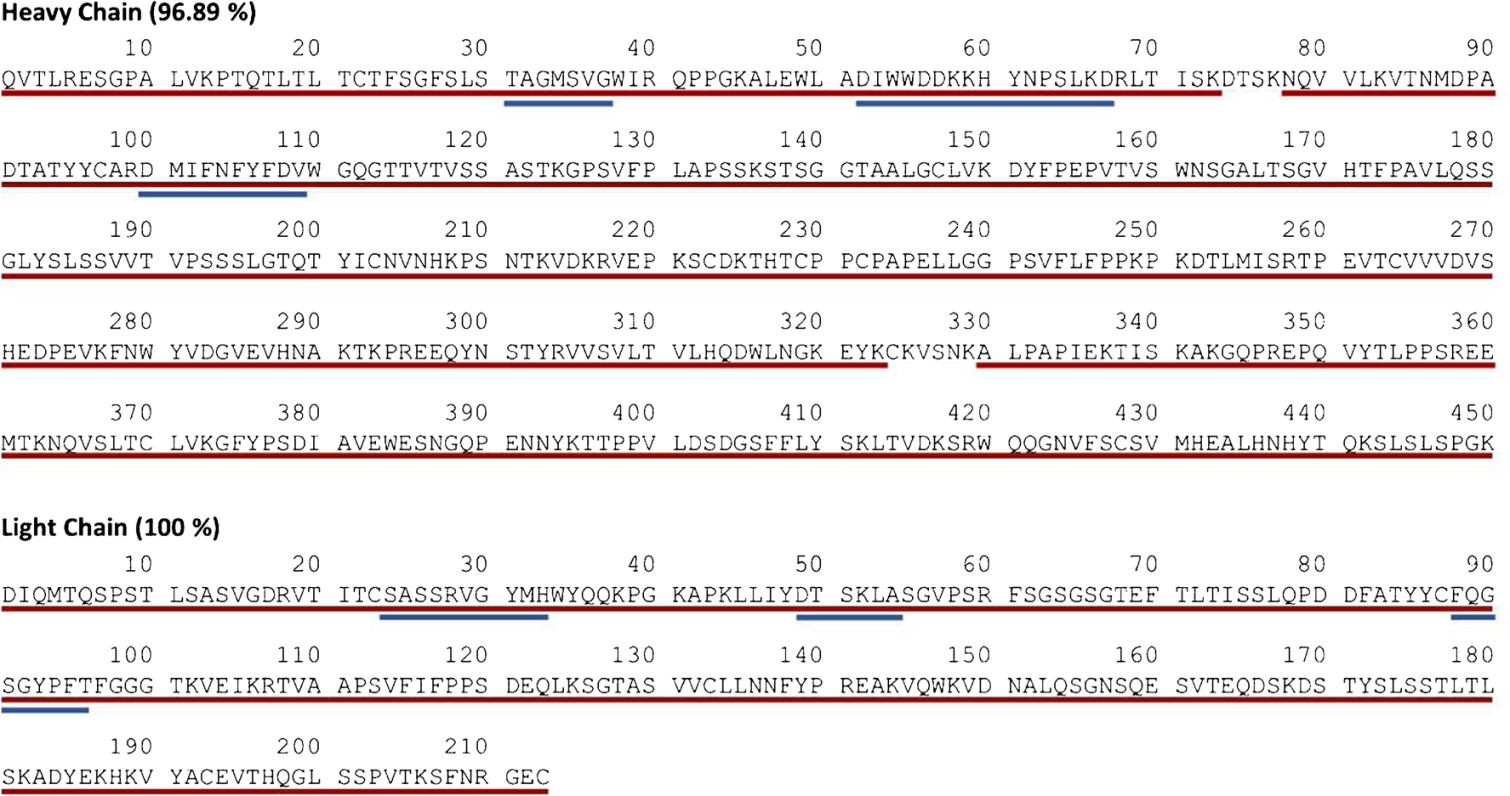
PS 8670 sequence coverage. Sequence coverage of the heavy and light chains of PS 8670 was calculated after identification of peptides detected in the peptide map. The amino acid sequence is shown with red underlining to indicate the identified regions. Blue underlining indicates CDRs

Following peptide identification, we calculated sequence coverage of the heavy chain to be 96.89% and to be 100% for the light chain (Fig. 8), which included full coverage of the complementarity-determining regions. Several peptides originating from trypsin autolysis were identified in the reference digest, but none from host cell proteins were detected. The peak list provided herein as ESM Table S2 was produced as a means of confirming the sequence of PS 8670 and subsequently RM 8671. This is by no means an exhaustive resource of the low abundant variations (e.g. post-translational modifications, sequence variants, glycoforms) and impurities (e.g. host cell proteins) that comprise the heterogeneity of the NISTmAb. Many of these attributes have been previously described (e.g. [48–51]) and undoubtedly there are additional variants yet to be identified. Indeed the continued exploration of the NISTmAb using alternate mass spectrometry instrumentation, data acquisition methods, analytical columns and data processing algorithms will tease out subtle attributes yet unknown. Such exercises will allow us to further probe the depth of our analytical capabilities, identify gaps and develop new technologies and methods to fill them.

#### Establishment of the identity of NISTmAb RM 8671

**Fig. 9:**
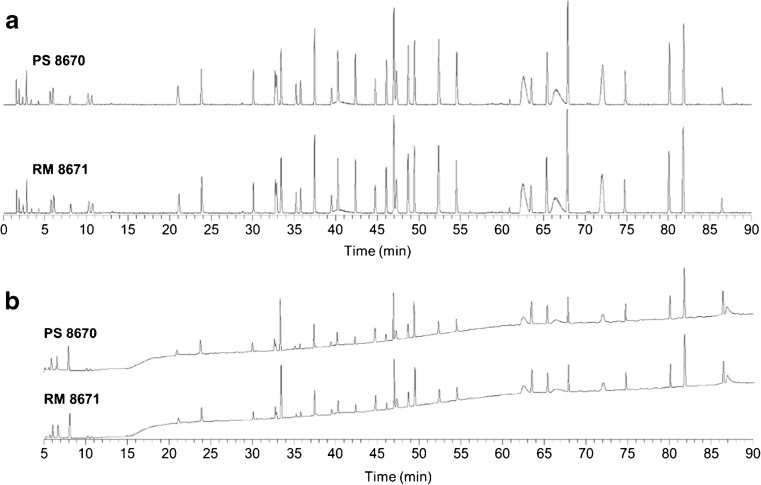
Alignment of PS 8670 with RM 8671. Tryptic digests of PS 8670 and RM 8671 were analyzed by LC-UV-MS and the resulting (**a**) TIC and (**b**) UV chromatograms compared. The initial five minutes of the UV traces are not shown due to the large difference in scale between the relative levels of absorbance of peaks detected during the 0 min to 5 min period and the 5 min to 90 min period. TIC and UV retention times are listed in ESM Table S3 and ESM Table S4, respectively. Corresponding peptide identifications are given in ESM Table S2

We used the newly generated PS 8670 peptide map to confirm that NISTmAb RM 8671 lot 14HB-D-002 (RM 8671) primary structure conforms to that of PS 8670. PS 8670 and RM 8671 were subjected to tryptic digestion per our optimized tryptic digest protocol (ESM Document S1). Both TIC and UV chromatograms of the digests were established by LC-UV-MS analyses. Alignment of the TIC and UV traces of the RM 8671 digest with the PS 8670 peptide map showed a high degree of sameness upon visual inspection (Fig. 9). No trace had a unique or missing peak as compared to the reference map with our criterium that TIC peaks have a minimum peak area > 0.5% of the area of the sum of all peaks.

Mean TIC retention times were calculated across quadruplicate injections of the RM 8670 digest (ESM Table S3) and data from three of the injections were used to calculate mean UV retention times (ESM Table S4). The difference between means of the reference map peak retention times and the corresponding peaks in the RM 8670 map was <2% for TIC and UV chromatograms, indicating conformity between the reference map and the RM.

To further confirm the identity of NISTmAb RM 8671, data from its tryptic digest were submitted for peptide identification using Byonic. Calculation of the sequence coverage for each tryptic digest produced the same results as those described for PS 8670 and shown in Fig. 8. The conformity of the TIC and UV peptide maps of PS 8670 and RM 8671 as well as the matching peptide identifications confirmed the identity of RM 8671.

## Conclusions

The NISTmAb is the first open access material available to the biopharmaceutical industry as a collaborative tool to promote the development of innovative methodology and technology. Its utility is strengthened by the accessibility of a comprehensive body of characterization data and specific protocols. The digestion method described in this manuscript is developed for use in conjunction with the NISTmAb and may serve as a “standard” digest when evaluating new analytical peptide mapping technologies and/or comparing innovative digestion protocols. A common digestion protocol will harmonize, and at least partially control, a large source of variation (i.e. the digestion) such that we can more readily delve into the variation resulting from the mass spectrometry instrumentation itself.

In developing this tryptic digestion protocol we used a step-wise approach to minimize modifications induced by the method while maximizing digestion efficiency. We chose to use this methodology because there is no single metric that defines a digest as optimal. In part this is due to the number of parameters that must be considered and the fact that one set of conditions may be optimal when focusing on one parameter, but are not optimal not for another. After reviewing the body of data as a whole we were able to filter out certain digest conditions as sub-optimal and identify one set of parameters that best fit our purpose. The conditions we chose provided minimal levels of artificially induced modifications and optimal sequence coverage when used to establish a peptide map of PS 8670. The sequence coverage obtained included full coverage of the CDR peptides, which gave us high confidence in confirming the primary structure of PS 8670. The peptide map generated using our optimized conditions was applied as an identity test for characterization of RM 8671. RM 8671 lot 14HB-D-001 was shown to visually conform to the PS 8670 reference map with no new or missing peaks. Peptide identifications made using MS/MS data as well as orthogonal techniques described in the accompanying studies in this issue [33, 52–54] further support the shared identity of RM 8671 with the PS 8670.

## Electronic supplementary material

ESM 1 (PDF 4.25 mb)
