# Supplementary material for: Development of an LC-MS/MS peptide mapping protocol for the NISTmAb
Source: Anal Bioanal Chem. 2018 Feb 7;410(8):2111–26. doi: 10.1007/s00216-018-0848-6 (PMC5830484; doi:10.1007/s00216-018-0848-6)
Supplement: Supplementary file 1 — (PDF 4.25 mb) [file 216_2018_848_MOESM1_ESM.pdf]

Analytical and Bioanalytical Chemistry

Electronic Supplementary Material

Development of an LC-MS/MS Peptide Mapping Protocol for the NISTmAb

Trina Mouchahoir, John E. Schiel

Document S1

Detailed protocol for optimized tryptic digestion of the NISTmAb

Buffer Preparation. Mass measurements for solution preparation were performed using a Mettler-Toledo AL54 analytical balance (calibrated annually). PH measurements were read using the Orion Star™ A211 pH Meter (Thermo Scientific) fitted with the ROSS Sure Flow Combination pH Electrode (Thermo Scientific). Protein concentration was measured using the Thermo Scientific™ NanoDrop 2000 spectrophotometer. Details regarding the reagents used can be found in the Materials and Methods section of the main manuscript.

The 0.1 mol/L Tris, pH 7.8 solution was prepared as follows: 1) 2.127 g Tris-HCl and 0.7877 g Tris base were added to a 200 mL volumetric flask; 2) the flask was filled to the 200.0 mL mark with LC-MS grade water; 3) while recording pH with a calibrated pH meter, the solution was adjusted to pH 7.8 ± 0.02 by drop-wise addition of 1 mol/L hydrochloric acid; 4) the solution was sterile filtered through a 0.22 µm cellulose acetate membrane; and 5) stored at 2 °C to 8 °C until use.

The 10 mmol/L EDTA in 0.1 mol/L Tris, pH 7.8 solution was prepared as follows: 1) 29.2 mg ethylenediaminetetraacetic acid (EDTA) were added to a 10 mL volumetric flask; 2) the flask was filled to the 10 mL mark with 0.1 mol/L Tris, pH 7.8; 3) the solution was sterile filtered through a 0.22 µm cellulose acetate membrane; and 5) stored at 2 °C to 8 °C until use.

Denaturing Buffer (6 mol/L guanidine HCl, 1 mmol/L EDTA in 0.1 mol/L Tris, pH 7.8) was prepared as follows: 1) 5.732 g guanidine HCl and 1 mL of 10 mmol/L EDTA were added to a 10 mL volumetric flask; 2) the flask was filled to 10 mL with 0.1 mol/L Tris, pH 7.8; 3) the solution was sterile filtered through a 0.22 µm cellulose acetate membrane; and 5) stored at 2 °C to 8 °C until use.

Formulation buffer (12.5 mmol/L L-histidine/12.5 mmol/L L-histidine HCl, pH 6.0) was prepared as follows: 1) 1.3129 g histidine monohydrochloride monohydrate and 0.9704 g L-histidine were placed in a beaker and diluted with ~450 mL LC-MS grade water; 2) while recording pH with a calibrated pH meter, the solution was adjusted to pH 6.00 ± 0.02 by drop-wise addition of 1 mol/L hydrochloric acid; 3) the solution was transferred to a 500 mL volumetric flask, the beaker rinsed with LC-MS grade water and the flask volume was adjusted

to 500.0 mL using the rinse water; 4) the solution was sterile filtered through a 0.22 µm cellulose acetate membrane into a sterile plastic bottle; and 5) stored at 2 °C to 8 °C.

500 mmol/L DTT was prepared as follows: 1) the tube containing 7.7 mg of dithiothreitol (DTT) (pre-weighed by manufacturer; Pierce No-Weigh Format DTT Cat #20291) was briefly centrifuged to bring all particles to the bottom; 2) 100 µL of LC-MS grade water were added to the tube; 3) the contents of the tube were mixed by gentle pipetting; and 3) the solution was stored in small volume aliquots at -80 °C.

500 mmol/L IAM was prepared as follows: 1) the tube containing 56 mg of iodoacetamide (IAM) (pre-weighed by manufacturer; Sigma pre-weighed vials, Cat #A3221) was briefly centrifuged to bring all particles to the bottom; 2) 600 µL of LC-MS grade water were added to the tube; 3) the contents of the tube were mixed by gentle pipetting; and 4) the solution was stored in small volume aliquots at -80 °C.

Digestion Buffer (1 mol/L urea in 0.1 mol/L Tris, pH 7.8) was prepared at the time of use as follows: 1) 300.3 mg urea were added to a 5 mL volumetric flask; 2) the flask was filled to 5 mL with 0.1 mol/L Tris, pH 7.8; and 3) the solution was sterile filtered through a 0.22 µm cellulose acetate membrane.

0.05 mol/L Acetic Acid was prepared as follows: 1) ≈ 25 mL of LC-MS grade water were added to a 25 mL volumetric flask; 2) 0.072 mL acetic acid (99.9% purity) were added to the flask while under a fume hood, using a glass syringe with stainless steel needle and polytetrafluoroethylene (PTFE) plunger; 3) the flask was filled to the 25 mL mark with LC-MS grade water; 4) the solution was sterile filtered through a 0.22 µm PTFE membrane into an amber glass reagent bottle with PTFE-lined cap; and 5) stored at 2 °C to 8 °C.

1 µg/µL Trypsin was prepared as follows: 1) 25 µL of 0.05 mol/L acetic acid were added to a 25 µg vial of trypsin (Recombinant, proteomics grade, expressed in *Pichia pastoris*; Sigma Cat # 03708985001); 2) the solution was mixed by gentle pipetting; and 3) stored in small volume aliquots at -80 °C.

0.1 % formic acid in LC-MS grade water was purchased as a pre-formulated solution.

Optimized Tryptic Digestion Protocol. The optimized tryptic digestion protocol is summarized below. The protocol begins with denaturing, reduction and alkylation of 500 µg of NISTmAb, and proceeds with the digestion of 50 µg of the reduced and alkylated NISTmAb. The buffer exchange and digestion (Step 4 through Step 6) may be scaled up to use a larger amount of reduced and alkylated NISTmAb if desired.

1. Sample and Blank Preparation
    a. 50 µL of 10 mg/mL NISTmAb were added to 445 µL Denaturing Buffer in a polypropylene tube
    b. in a separate tube, a blank was prepared by adding 50 µL of Formulation Buffer to 445 µL Denaturing Buffer
2. Reduction
    a. 5 µL of 500 mmol/L DTT were added to each tube
    b. the contents of each tube were mixed gently
    c. the tubes were incubated at 4°C for 60 min, in the dark
3. Alkylation
    a. 10.2 µL of 500 mmol/L IAM were added to each tube
    b. the contents of each tube were mixed gently
    c. the tubes were incubated at 4°C for 60 min, in the dark
4. Buffer Exchange (using Zeba™ Spin 7K MWCO size-exclusion desalting columns (P/N 89882, Thermo Scientific, Waltham, MA))
    a. fresh Digestion Buffer was made and used to prepare two size exclusion spin columns for buffer exchange according to the manufacturer's specifications
    b. after spin columns were exchanged to Digestion Buffer and the alkylation incubation period was complete the columns were placed in clean, labeled 1.5 mL polypropylene tubes
    c. 100 µL of the reduced and alkylated NISTmAb were added to the center of one column bed without disturbing the bed with the pipet tip
    d. 100 µL of the blank were added to the center of the remaining column bed
    e. the columns were centrifuged according to the manufacturer's specifications to collect the sample and blank which were then in Digestion Buffer
5. Protein Concentration
    a. the contents of the buffer-exchanged blank tube were used to "blank" the spectrophotometer
    b. the protein concentration of the buffer-exchanged NISTmAb was measured by UV-VIS at 280 nm absorbance using the manufacturer default settings for IgG

6. Digestion
   a. the measured protein concentration was used to determine the volume needed for 50 µg of buffer-exchanged NISTmAb and this volume was transferred to a new polypropylene tube:

$$\text{Volume of NISTmAb needed } (\mu\text{L}) = \frac{50\ \mu g}{concentration\ (\mu g/\mu L)}$$

   b. 2.78 µL of 1 µg/µL trypsin were added to the 50 µg aliquot of NISTmAb (a 1:18 (w/w) enzyme:NISTmAb ratio)
   c. the volume of Digestion Buffer needed to dilute the sample to 0.5 µg/µL was determined:

$$Volume\ of\ Digestion\ Buffer\ needed\ (\mu L)\ =\ final\ volume - volume\ of\ IgG\ added - trypsin\ volume$$

$$Volume\ of\ Digestion\ Buffer\ needed\ (\mu L)\ =\ 100\ \mu L - volume\ of\ IgG\ added\ (\mu L) - 2.78\ \mu L$$

   d. the calculated volume of Digestion Buffer was added to the sample tube and the contents were mixed gently
   e. the sample was incubated at room temperature for 4 hr
   f. 100 µL of 0.1 % formic acid in LC-MS grade water was added to stop the digestion, bringing the final NISTmAb concentration to 0.25 µg/µL
   g. the digested NISTmAb was stored at -80 °C until analysis

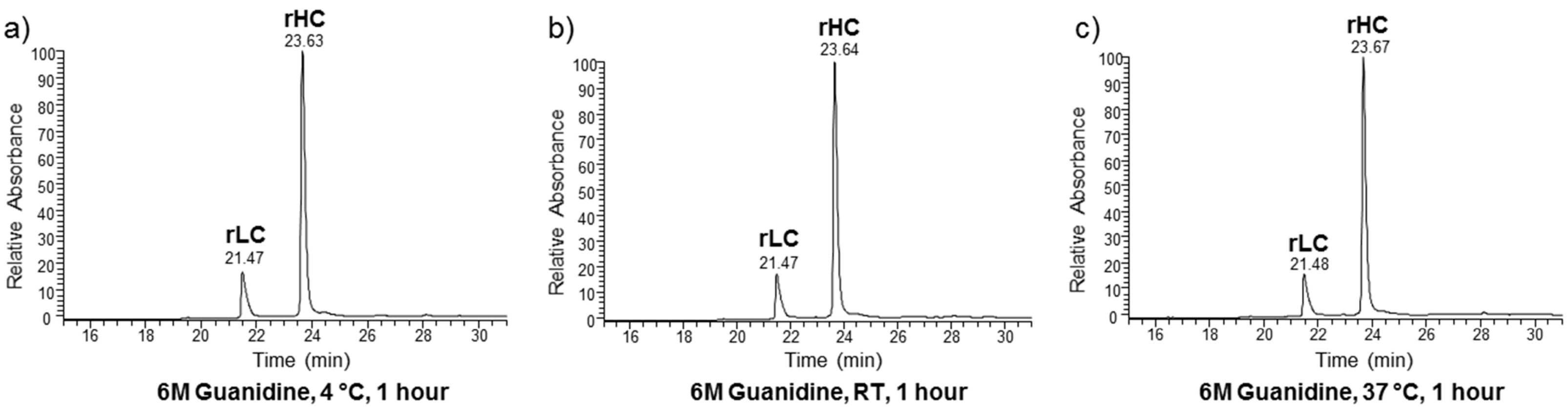

Fig. S1 LC-UV chromatograms of IgG reduced at various temperatures PS 8670 was incubated in 6.0 mol/L guanidine HCl buffer with 20 mmol/L DTT for 1 hr at a) 4 °C; b) room temperature (RT); or c) 37 °C. The level of reduction was determined by LC-UV-MS analysis of 5 µg of mAb. The species comprising each UV peak were identified by deconvolution of their corresponding MS spectra. LC = light chain; HC = heavy chain; r = reduced. Representative deconvoluted masses calculated for each species are listed in ESM Table S1.

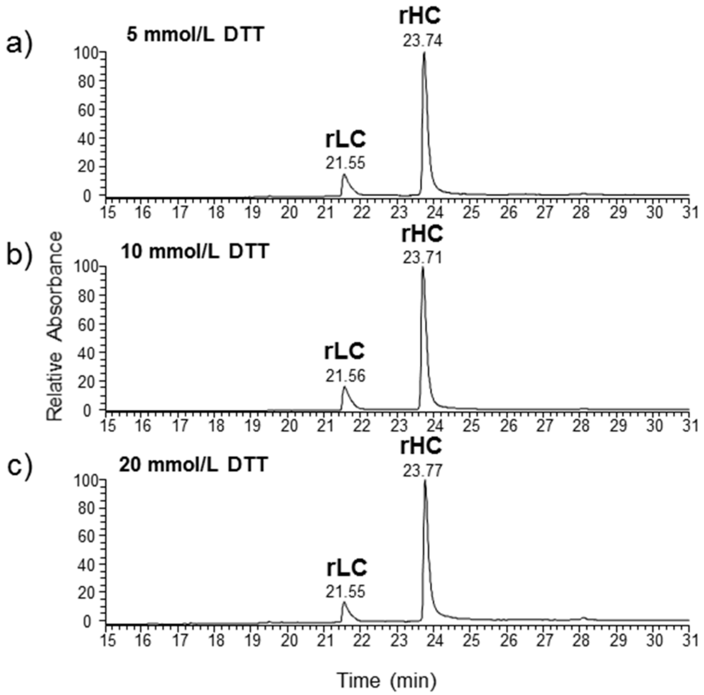

Fig. S2 LC-UV chromatograms of IgG reduced at various DTT concentrations PS 8670 was reduced at 4 °C for 1 hr in 6.0 mol/L guanidine HCl buffer and either a) 5 mmol/L DTT; b) 10 mmol/L DTT; or c) 20 mmol/L DTT. The level of reduction was determined by LC-UV-MS analysis of 5 µg of mAb. The species comprising each UV peak were identified by deconvolution of their corresponding MS spectra. LC = light chain; HC = heavy chain; r = reduced. Representative deconvoluted masses calculated for each species are listed in ESM Table S1.

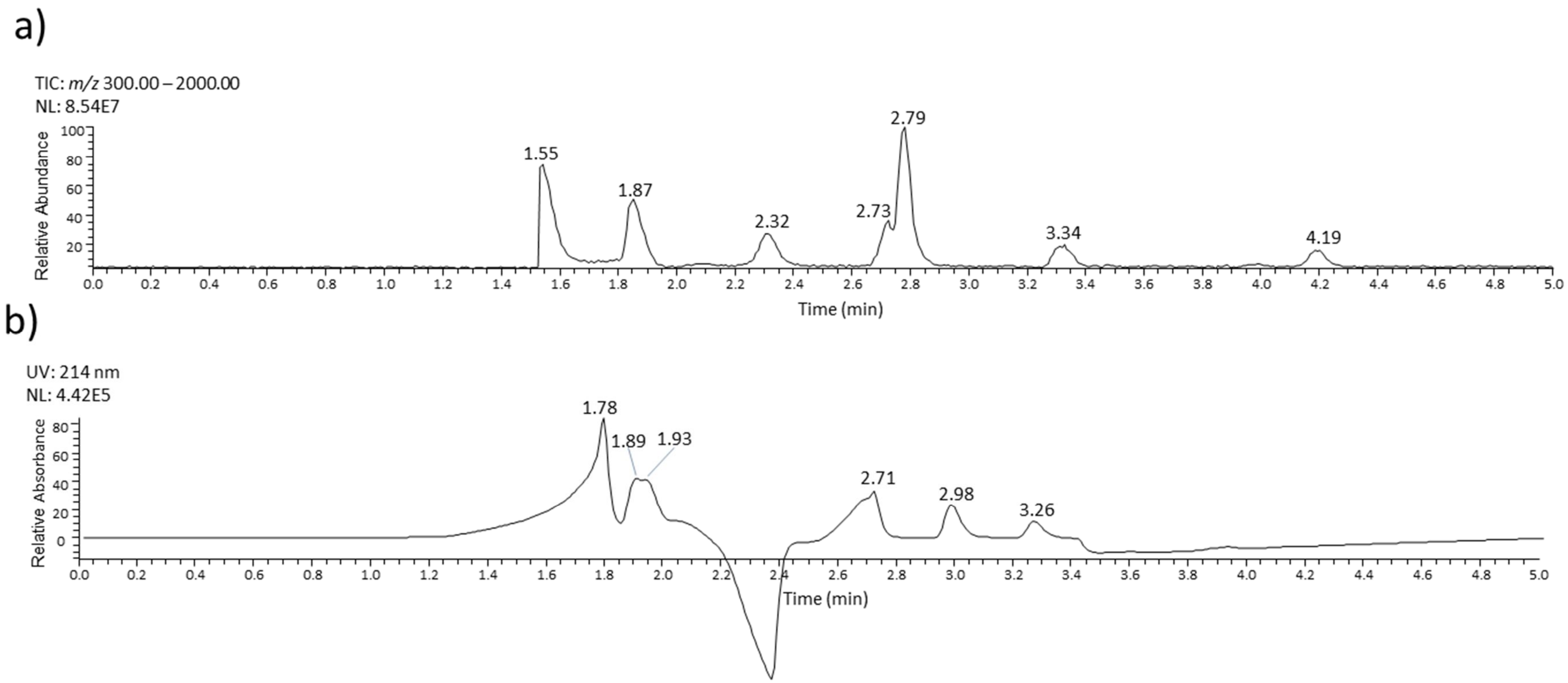

Fig. S3 Zoomed view of PS 8670 peptide map (0 min to 5.0 min) PS 8670 tryptic digest was analyzed by LC-UV-MS in quadruplicate. Mean retention times for TIC and UV chromatographic peaks were calculated as shown in ESM Table S2. Those values are used here to label their corresponding peaks (panel a = TIC; panel b = UV 214 nm) and together constitute the peptide map.

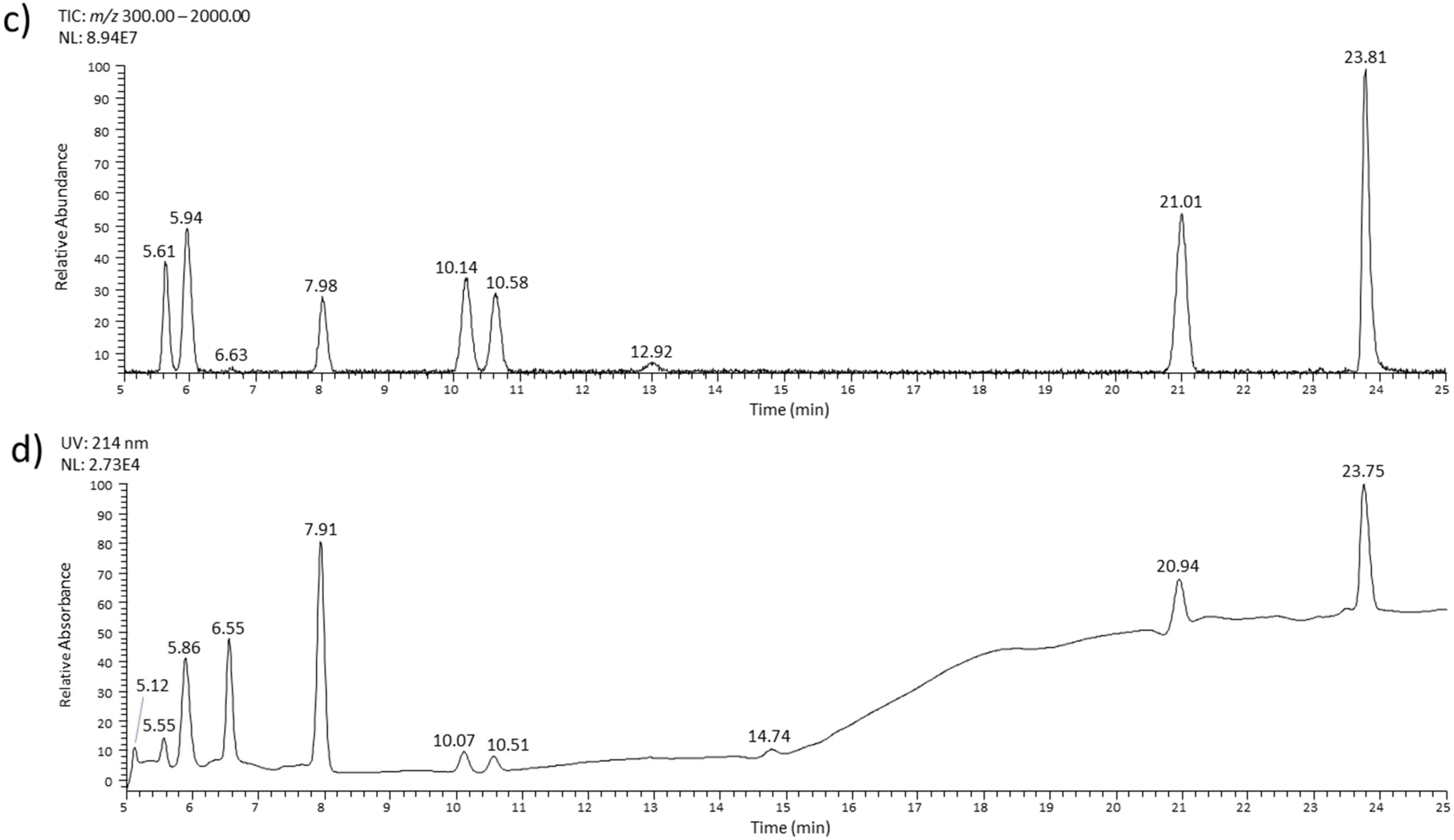

Fig. S3 (continued) Zoomed view of PS 8670 peptide map (5.0 min to 25.0 min) PS 8670 tryptic digest was analyzed by LC-UV-MS in quadruplicate. Mean retention times for TIC and UV chromatographic peaks were calculated as shown in ESM Table S2. Those values are used here to label their corresponding peaks (panel c = TIC; panel d = UV 214 nm) and together constitute the peptide map.

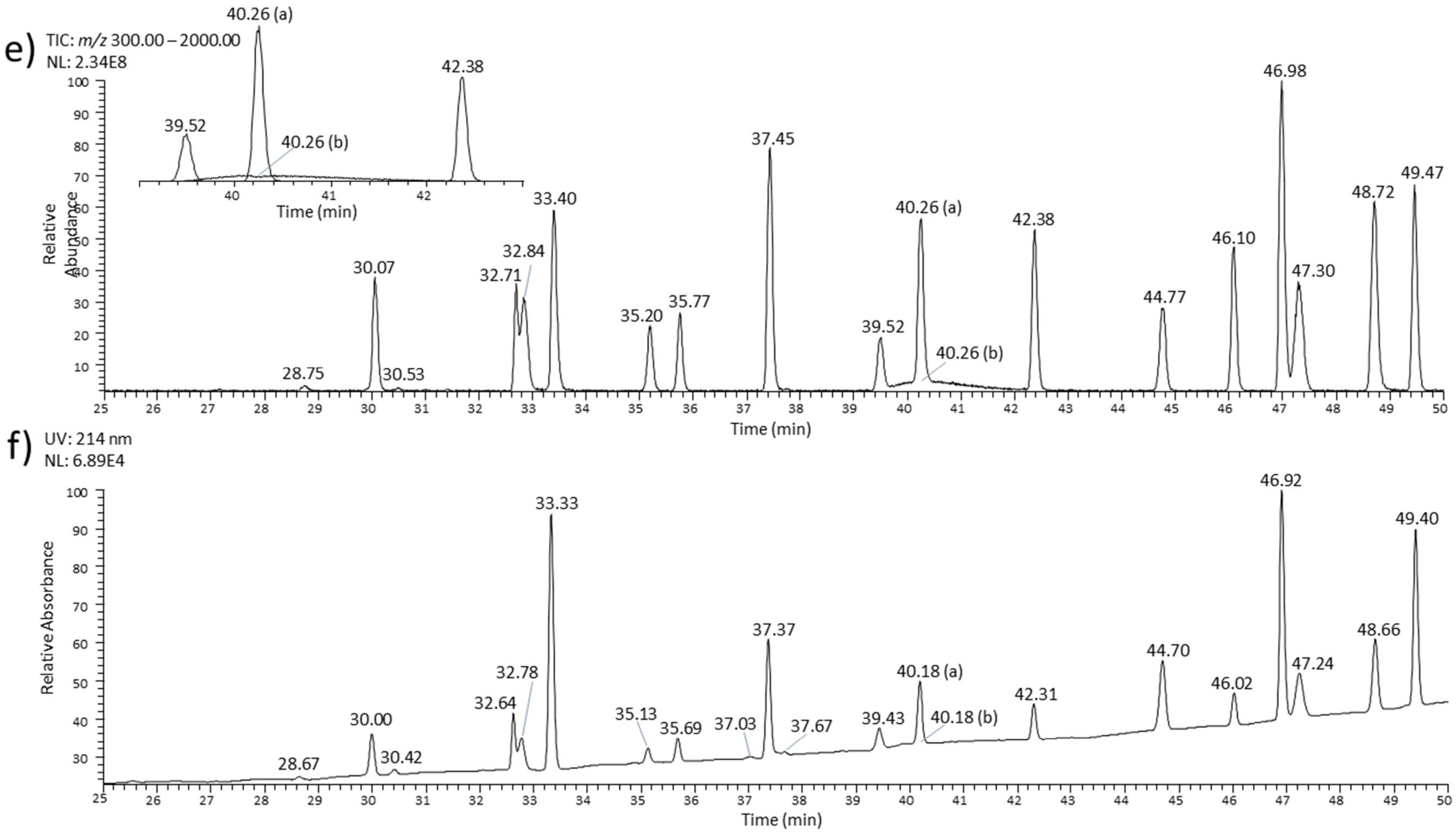

Fig. S3 (continued) Zoomed view of PS 8670 peptide map (25.0 min to 50.0 min) PS 8670 tryptic digest was analyzed by LC-UV-MS in quadruplicate. Mean retention times for TIC and UV chromatographic peaks were calculated as shown in ESM Table S2. Those values are used here to label their corresponding peaks (panel e = TIC; panel f = UV 214 nm) and together constitute the peptide map. The species eluting at RT 40.26 min form two distinct peaks in the TIC trace. These were manually determined to share the same apex and are therefore designated with RT 40.26 (a) and RT 40.26 (b). The panel (e) inset overlays extracted ion chromatograms (XICs) for peptides eluting between RT 39.0 min and 43.0 min to highlight the presence of distinct peaks at RT 40.26 min.

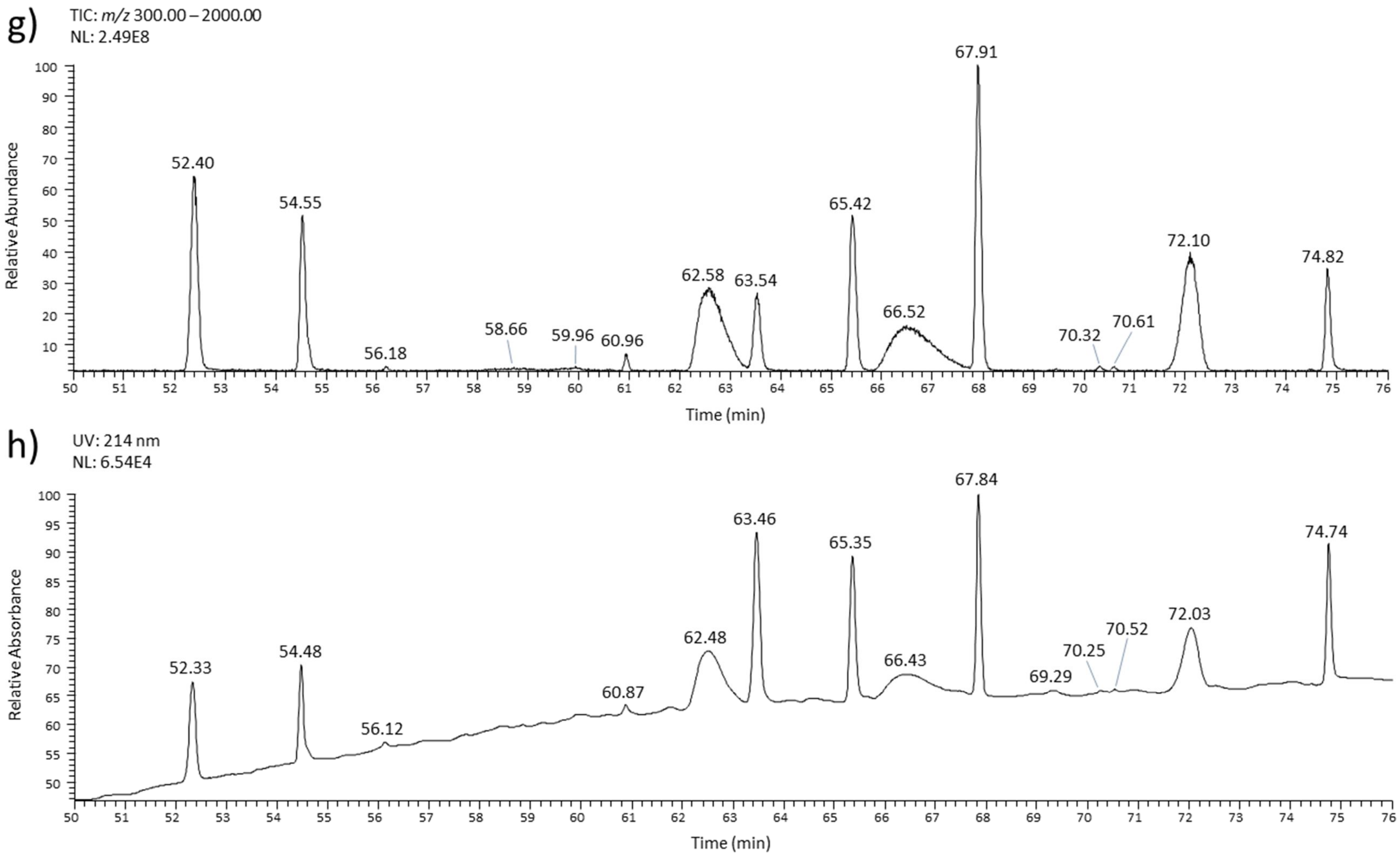

Fig. S3 (continued) Zoomed view of PS 8670 peptide map (50.0 min to 76.0 min) PS 8670 tryptic digest was analyzed by LC-UV-MS in quadruplicate. Mean retention times for TIC and UV chromatographic peaks were calculated as shown in ESM Table S2. Those values are used here to label their corresponding peaks (panel g = TIC; panel h = UV 214 nm) and together constitute the peptide map.

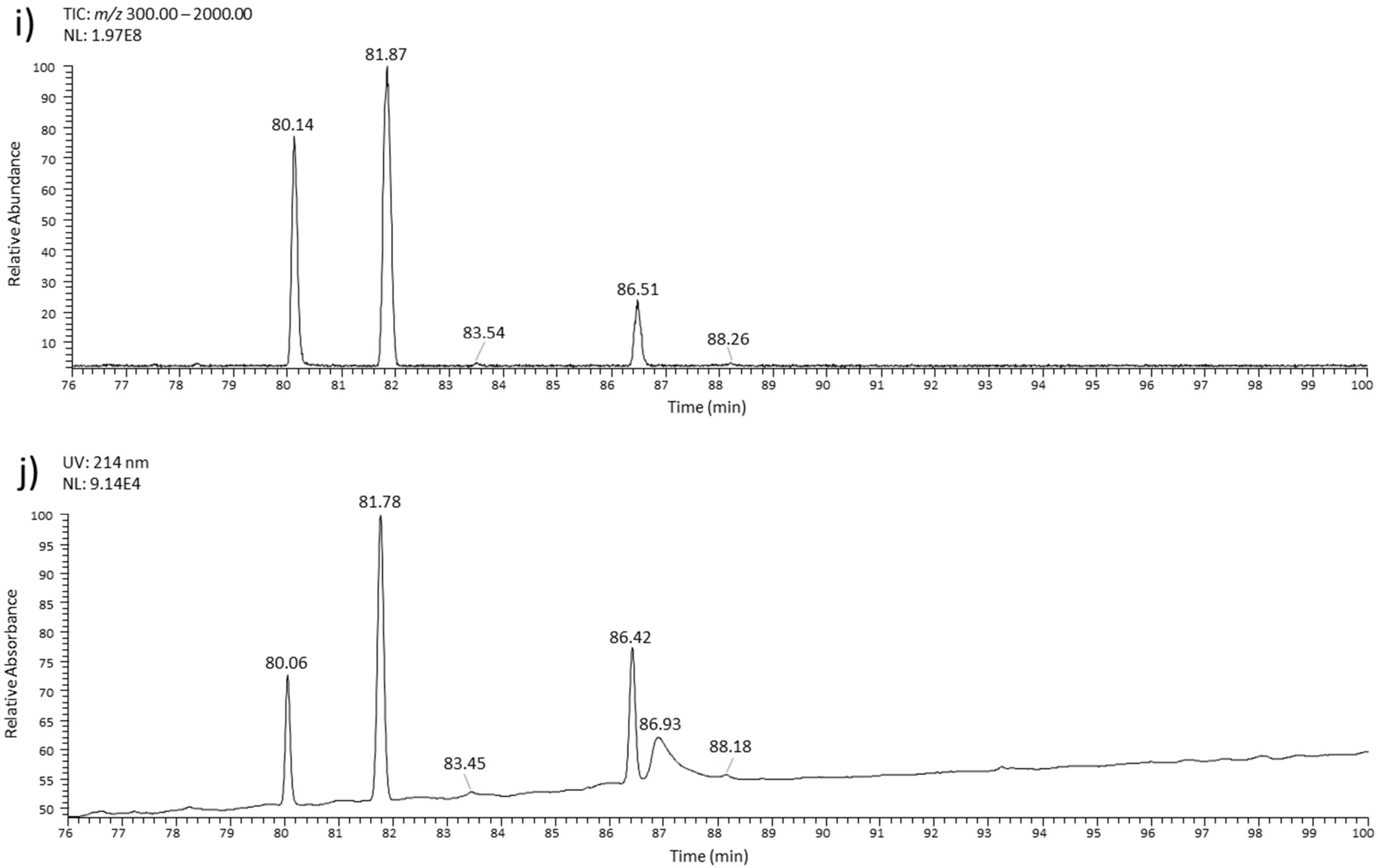

Fig. S3 (continued) Zoomed view of PS 8670 peptide map (76.0 min to 100.0 min) PS 8670 tryptic digest was analyzed by LC-UV-MS in quadruplicate. Mean retention times for TIC and UV chromatographic peaks were calculated as shown in ESM Table S2. Those values are used here to label their corresponding peaks (panel i = TIC; panel j = UV 214 nm) and together constitute the peptide map.

Table S1 Representative deconvoluted masses of PS 8670 subunit peaks The species comprising each UV peak shown in Fig. 1, Fig. 2, Fig. 3, ESM Fig. S1 and ESM Fig. S2 were identified by deconvolution of their corresponding MS spectra. Observed and calculated average masses representative of each species are shown. LC = light chain; HC = heavy chain.

Reduced and Partially Reduced Species

Peak Label	Species	Intact Disulfide bonds	Observed Average Mass (Da)	Calculated Average Mass (Da)	error (ppm)
prLC (2)	Partially reduced LC	2	23123.5550	23123.4776	3.35
rLC	Fully reduced LC	0	23127.5430	23127.5093	1.46
prHC (3)	Partially reduced HC (G0F)	3	50900.6090	50900.7752	-3.27
prHC (3)	Partially reduced HC (G1F)	3	51062.0430	51062.9158	-17.09
prHC (4)	Partially reduced HC (G0F)	4	50897.8390	50898.7594	-18.08
prHC (4)	Partially reduced HC (G1F)	4	51059.9610	51060.9000	-18.39
prHC (5)	Partially reduced HC (G0F)	5	50898.2420	50898.7594	-10.17
prHC (5)	Partially reduced HC (G1F)	5	51057.2850	51058.8841	-31.32
rHC	Fully Reduced HC (G0F)	0	50906.4610	50906.8229	-7.11
rHC	Fully Reduced HC (G1F)	0	51068.6990	51068.9635	-5.18
prLC+HC (6)	Partially reduced LC + HC (G0F)	6	74022.0700	74022.2369	-2.25
prLC+HC (6)	Partially reduced LC + HC (G1F)	6	74184.6880	74184.3775	4.19
prLC+HC (7)	Partially reduced LC + HC (G0F)	7	74020.7730	74020.2211	7.46

Fully Reduced and Alkylated Species

Peak Label	Species	Alkylated Cysteines	Observed Average Mass (Da)	Calculated Average Mass (Da)	error (ppm)
aLC	Fully Reduced and Alkylated LC	5	23412.91	23412.7658	6.16
aHC	Fully Reduced and Alkylated HC (G0F)	11	51534.996	51534.3872	11.81
aHC	Fully Reduced and Alkylated HC (G1F)	11	51696.93	51696.5278	7.78

Table S2 Identification of peptides contributing to TIC and UV chromatogram peaks Peptides generated from tryptic digestion of PS 8670 were analyzed by LC-UV-MS. TIC and UV peaks reported in Figure 7 and ESM Figure S3 were correlated with the peptide identifications. Lower case letters in the peptide sequence reflect the location of the observed modification. nd = not detected. Peptide location is described as "H" for Heavy Chain or "L" for Light Chain, followed by the first and last residue numbers relative to the N-terminal residue of that chain.

<table>
<tr><th>Mean TIC RT (min)</th><th>Mean UV RT (min)</th><th>Peptide</th><th>Variable Modification (Residue)</th><th>Peptide Location</th><th>Obs. MW (uncharged)</th><th>Calc. MW (uncharged)</th><th>Charge States Observed (z)</th><th>Comment</th></tr>
<tr><td>1.55</td><td>nd</td><td>(Buffer component)</td><td></td><td></td><td></td><td></td><td></td><td></td></tr>
<tr><td rowspan="12">1.87</td><td rowspan="12">1.78</td><td>TISK</td><td></td><td>H(338-341)</td><td>447.2693</td><td>447.2690</td><td>1</td><td rowspan="12">Predominant ions are unidentified (they are most likely buffer components)</td></tr>
<tr><td>APK</td><td></td><td>L(42-44)</td><td>314.1957</td><td>314.1954</td><td>1</td></tr>
<tr><td>QPPGK</td><td></td><td>H(41-45)</td><td>525.2912</td><td>525.2911</td><td>1</td></tr>
<tr><td>VEPK</td><td></td><td>H(218-221)</td><td>471.2696</td><td>471.2693</td><td>1</td></tr>
<tr><td>GQPR</td><td></td><td>H(344-347)</td><td>456.2444</td><td>456.2445</td><td>1</td></tr>
<tr><td>VDKR</td><td></td><td>H(214-217)</td><td>516.3018</td><td>516.3020</td><td>1</td></tr>
<tr><td>EAK</td><td></td><td>L(142-144)</td><td>346.1848</td><td>346.1850</td><td>1</td></tr>
<tr><td>EYK</td><td></td><td>H(321-323)</td><td>438.2110</td><td>438.2114</td><td>1</td></tr>
<tr><td>SCDK</td><td></td><td>H(222-225)</td><td>508.1952</td><td>508.1950</td><td>1</td></tr>
<tr><td>GEC</td><td></td><td>L(211-213)</td><td>364.1057</td><td>364.1050</td><td>1</td></tr>
<tr><td>RVEPK</td><td></td><td>H(217-221)</td><td>627.3731</td><td>627.3700</td><td>2</td></tr>
<tr><td>TKPR</td><td></td><td>H(292-295)</td><td>500.3072</td><td>500.3071</td><td>1</td></tr>
<tr><td>nd</td><td>1.89</td><td>(Buffer component)</td><td></td><td></td><td></td><td></td><td></td><td></td></tr>
<tr><td>nd</td><td>1.93</td><td>(Buffer component)</td><td></td><td></td><td></td><td></td><td></td><td></td></tr>
<tr><td>2.32</td><td>nd</td><td>EEMTK</td><td></td><td>H(359-363)</td><td>636.2788</td><td>636.2789</td><td>1</td><td></td></tr>
<tr><td>2.73</td><td>nd</td><td>ADYEK</td><td></td><td>L(183-187)</td><td>624.2756</td><td>624.2757</td><td>1</td><td></td></tr>
<tr><td rowspan="4">2.79</td><td rowspan="4">2.71</td><td>SFNR</td><td></td><td>L(207-210)</td><td>522.2550</td><td>522.2550</td><td>1</td><td rowspan="4">ADYEK peptide identified with substantial intensity from unresolved peak with TIC apex at RT 2.73 min.</td></tr>
<tr><td>VEIKR</td><td></td><td>L(103-107)</td><td>643.4027</td><td>643.4017</td><td>2</td></tr>
<tr><td>KHYNPSLK</td><td></td><td>H(59-66)</td><td>985.5344</td><td>985.5340</td><td>2</td></tr>
<tr><td>NKPGVYTK (Trypsin)</td><td></td><td></td><td>905.4980</td><td>905.4971</td><td>2</td></tr>
<tr><td>nd</td><td>2.98</td><td>(Buffer component)</td><td></td><td></td><td></td><td></td><td></td><td></td></tr>
<tr><td>3.34</td><td>3.26</td><td>(Buffer component)</td><td></td><td></td><td></td><td></td><td></td><td></td></tr>
<tr><td>4.19</td><td>nd</td><td>VEIK</td><td></td><td>L(103-106)</td><td>487.3005</td><td>487.3010</td><td>1</td><td></td></tr>
<tr><td>nd</td><td>5.12</td><td>Not determined</td><td></td><td></td><td></td><td></td><td></td><td></td></tr>
<tr><td>5.61</td><td>5.55</td><td>LTVDK</td><td></td><td>H(413-417)</td><td>574.3328</td><td>574.3326</td><td>1</td><td></td></tr>
<tr><td rowspan="2">5.94</td><td rowspan="2">5.86</td><td>HYNPSLK</td><td></td><td>H(60-66)</td><td>857.4393</td><td>857.4395</td><td>1,2</td><td rowspan="2"></td></tr>
<tr><td>WYQQKPGK</td><td></td><td>L(34-41)</td><td>1033.5349</td><td>1033.5345</td><td>2</td></tr>
<tr><td>nd</td><td>6.55</td><td>(Buffer component)</td><td></td><td></td><td></td><td></td><td></td><td></td></tr>
<tr><td>7.98</td><td>7.91</td><td>VQWK</td><td></td><td>L(145-148)</td><td></td><td>559.3120</td><td>1</td><td></td></tr>
<tr><td>10.14</td><td>10.07</td><td>NQVVLK</td><td></td><td>H(78-83)</td><td></td><td></td><td>1,2</td><td></td></tr>
<tr><td>10.58</td><td>10.51</td><td>LTISK</td><td></td><td>H(69-73)</td><td>560.3542</td><td>560.3530</td><td>1</td><td></td></tr>
<tr><td>12.92</td><td>nd</td><td>DRLTISK</td><td></td><td>H(67-73)</td><td>831.4815</td><td>831.4810</td><td>2</td><td></td></tr>
<tr><td>nd</td><td>14.74</td><td>(Buffer component)</td><td></td><td></td><td></td><td></td><td></td><td></td></tr>
<tr><td>21.01</td><td>20.94</td><td>LASGVPSR</td><td></td><td>L(53-60)</td><td>785.4400</td><td>785.4395</td><td>1,2</td><td></td></tr>
<tr><td rowspan="4">23.81</td><td rowspan="4">23.75</td><td>VTITCSASSR</td><td></td><td>L(19-28)</td><td>1080.5232</td><td>1080.5233</td><td>1,2</td><td rowspan="4"></td></tr>
<tr><td>EEQYnSTYR</td><td>G0F (N300)</td><td>H(296-304)</td><td>2429.9577</td><td>2633.0400</td><td>2</td></tr>
<tr><td>EEQYnSTYR</td><td>G1F (N300)</td><td>H(296-304)</td><td>2429.9577</td><td>2795.0900</td><td>2</td></tr>
<tr><td>EEQYnSTYR</td><td>G2F (N300)</td><td>H(296-304)</td><td>2429.9577</td><td>2957.1400</td><td>2</td></tr>
<tr><td rowspan="2">28.75</td><td rowspan="2">28.67</td><td>SLSLSPGK</td><td></td><td>H(443-450)</td><td>787.4442</td><td>787.4440</td><td>1,2</td><td></td></tr>
<tr><td>VGYmHWYQQKPGK</td><td>Oxidation (M34)</td><td>L(29-41)</td><td>1636.7758</td><td>1636.7800</td><td>3</td><td></td></tr>
<tr><td rowspan="2">30.07</td><td rowspan="2">30</td><td>HKVYACEVTHQGLSSPVTK</td><td></td><td>L(188-206)</td><td>2140.0765</td><td>2140.0735</td><td>2,3,4</td><td rowspan="2"></td></tr>
<tr><td>LSSPATLNSR (Trypsin)</td><td></td><td></td><td>1044.5565</td><td>1044.5564</td><td>1</td></tr>
<tr><td>30.53</td><td>30.42</td><td>VGYMHWYQQKPGKAPK</td><td></td><td>L(29-44)</td><td>1916.9731</td><td>1916.9719</td><td>3,4</td><td></td></tr>
<tr><td>32.71</td><td>32.64</td><td>VDNALQSGNSQESVTEQDSK</td><td></td><td>L(149-168)</td><td>2134.9626</td><td>2134.9614</td><td>2,3</td><td>ALPAPIEK peptide identified with trace intensity from unresolved peak with TIC apex at RT 32.84 min (UV RT 32.78 min).</td></tr>
</table>

Mean TIC RT (min)	Mean UV RT (min)	Peptide	Variable Modification (Residue)	Peptide Location	Obs. MW (uncharged)	Calc. MW (uncharged)	Charge States Observed (*z*)	Comment
32.84	32.78	ALPAPIEK		H(330-337)	837.4969	837.4960	1,2	VDNALQSGNSQESVTEQDSK peptide identified with minor intensity from unresolved peak with TIC apex at RT 32.71 min (UV RT 32.64 min).
33.4	33.33	VGYMHWYQQKPGK		L(29-41)	1620.7899	1620.7871	2,3,4	
35.2	35.13	qVTLR	Gln -> pyroGlu (Q1)	H(1-5)	598.3444	598.3440	1	
		VATVSLPR (Trypsin)			841.5020	841.5021	1,2	
35.77	35.69	DTLMISR		H(252-258)	834.4278	834.4269	1,2	
nd	37.03	Not determined						
37.45	37.37	VYACEVTHQGLSSPVTK		L(190-206)	1874.9228	1874.9196	2,3	
		VGYMHWYQQK		L(29-38)	1338.6194	1338.6179	2,3	
nd	37.67	Not determined						
39.52	39.43	SLSLSPGk	Lys-loss (K450)	H(443-450)	659.3493	659.3490	1	
		EPQVYTLPPSR		H(348-358)	1285.6683	1285.6666	2,3	Co-eluting peptide from front of TIC peak at RT 40.26 (b) (UV RT 40.18 (b)).
		TVLHQDWLNGK		H(310-320)	1309.6794	1309.6800	2,3	
40.26 (a)	40.18 (a)	LLIYDTSK		L(45-52)	951.5283	951.5277	1,2	This TIC peak is labeled as RT 40.26 (a) to distinguish from the broad peak having the same apex, but clearly distinct in shape, height and width from this peak. The broader peak is RT 40.26 (b).
		WQQGNVFSCSVmHEALHNHYTQK	Oxidation (M431)	H(420-442)	2816.2642	2816.2547	4	
40.26 (b)	nd	EPQVYTLPPSR		H(348-358)	1285.6683	1285.6666	2,3	Broad peak co-elutes with multiple other peaks. XIC indicates the apex of the TIC peak to be at RT 40.28 min. Because the peak is clearly distinct in shape, height and width from other species eluting at RT 40.26 min, it is distinguished as RT 40.26 (b). The TIC peak formed by the remaining species at this RT is distinguished as 40.26 (a).
42.38	42.31	STSGGTAALGCLVK		H(137-150)	1320.6725	1320.6707	1,2,3	
		DIQmTQSPSTLSASVGDR	Oxidation (M4)	L(1-18)	1907.8922	1907.8900	2,3	
44.77	44.7	VTNMDPADTATYYCAR		H(84-99)	1847.7834	1847.7818	2,3	
46.1	46.02	NQVSLTCLVK		H(364-373)	1160.6238	1160.6223	1,2	
46.98	46.92	WQQGNVFSCSVMHEALHNHYTQK		H(420-442)	2800.2638	2800.2598	2,3,4,5	
47.3	47.24	GPSVFPLAPSSK		H(125-136)	1185.6403	1185.6393	1,2	
48.72	48.66	DIQMTQSPSTLSASVGDR		L(1-18)	1891.8968	1891.8945	2,3	
		WQQGNVFSC		H(420-428)	1124.4701	1124.4700	1	
49.47	49.4	FNWYVDGVEVHNAK		H(278-291)	1676.7953	1676.7947	2,3	
52.4	52.33	TPEVTCVVVDVSHEDPEVK		H(259-277)	2138.0223	2138.0201	2,3	
54.55	54.48	DSTYSLSSTLTLSK		L(169-182)	1501.7541	1501.7512	1,2	
		nQVSLTCLVK	Deamidation (N364)	H(364-373)	1161.6086	1161.6100	2	
		LGEHNIDVLEGNEQFINAAK (Trypsin)			2210.1025	2210.0967	2,3	
56.18	56.12	SLSTAGMSVGWIR		H(28-40)	1363.6930	1363.6918	2	

Mean TIC RT (min)	Mean UV RT (min)	Peptide	Variable Modification (Residue)	Peptide Location	Obs. MW (uncharged)	Calc. MW (uncharged)	Charge States Observed (*z*)	Comment
58.66	nd	RTVAAPSVFIFPPSDEQLK		L(107-125)	2101.1241	2101.1207	2,3	
		GPSVFPLAPSSKSTSGGTAALGCLVK		H(125-150)	2488.3065	2488.3000	3	
		IITHPNFNGNTLDNDImLIK (Trypsin)	Oxidation (M94)		2281.1652	2298.1700	2,3	
59.96	nd	SCDKTHTCPPCPAPELLGGPSVFLFPPKPK		H(222-251)	3333.6394	3333.6348	4	RTVAAPSVFIFPPSDEQLK peptide identified with substantial intensity from unresolved peak with TIC apex at RT 58.66 min.
		FNWYVDGVEVH		H(278-288)	1363.6213	1363.6197	2	
60.96	60.87	IITHPNFNGNTLDNDIMLIK (Trypsin)			2282.1785	2282.1729	2,3	
		VVSVLTVLHQDwLNGK	Dioxidation (W316)	H(305-320)	1838.9882	1838.9900	2,3	
62.58	62.48	THTCPPCPAPELLGGPSVFLFPPKPK		H(226-251)	2843.4562	2843.4502	2,3,4	
		ESGPALVKPTQTLTLTCTF		H(6-24)	2063.0637	2063.0609	2	
		VVSVLTVLHQDWLNGKEYK		H(305-323)	2227.2041	2227.2001	3,4	
63.54	63.46	GFYPSDIAVEWESNGQPENNYK		H(374-395)	2543.1318	2543.1241	2,3	
65.42	65.35	TTPPVLDSDGSFFLYSK		H(396-412)	1872.9181	1872.9145	2,3	
66.52	66.43	TVAAPSVFIFPPSDEQLK		L(108-125)	1945.0244	1945.0196	2,3	
67.91	67.84	VVSVLTVLHQDWLNGK		H(305-320)	1807.0023	1806.9992	2,3	
		SGFSLSTAGMSVGWIR		H(25-40)	1654.8155	1654.8137	2	
nd	69.29	Not determined						
70.32	70.25	VVSVLTVLHQDWLnGK	Succinimide (N318)	H(305-320)	1789.9754	1789.9726	2,3	
70.61	70.52	ESGPALVKPTQTLTLTCTFSGF		H(6-27)	2354.1857	2354.1828	2	
72.1	72.03	SGTASVVCLLNNFYPR		L(126-141)	1796.8911	1796.8879	2,3	
74.82	74.74	ALEWLADIWWDDKK		H(46-59)	1787.8902	1787.8882	2,3	
		SLQPDDFATYYCFQGSGYPFTFGGGTK		L(76-102)	3010.3114	3010.3100	4	
		ESGPALVKPTQTLTLTCTFSGFSLSTAGMSVGWIRQPPGK		H(6-45)	4207.1485	4207.1400	4	
80.14	80.06	ESGPALVKPTQTLTLTCTFSGFSLSTAGMSVGWIR		H(6-40)	3699.8724	3699.8640	2,3,4	
81.87	81.78	DYFPEPVTVSWNSGALTSGVHTFPAVLQSSGLYSLSSVVTVPSSSLGTQTYICNVNHKPSNTK		H(151-213)	6712.3072	6712.3071	4,5	
83.54	83.45	ALEWLADIWWDDK		H(46-58)	1659.7953	1659.7933	2	
86.51	86.42	FSGSGSGTEFTLTISSLQPDDFATYYCFQGSGYPFTFGGGTK		L(61-102)	4481.9989	4481.9950	4	
nd	86.93	Trypsin digest component						Non-specific identification; this peak is also generated upon injection of mock digest (containing all components except mAb)
88.26	88.18	DMIFNFYFDVWGQGTTVTVSSASTK		H(100-124)	2800.3088	2800.3054	2,3	

Table S3 Comparison of TIC retention times between the PS 8670 reference map and RM 8671 Peptides generated from tryptic digestion of RM 8671 were analyzed by UV-LC-MS in quadruplicate. TIC peak retention times (RT) were extracted using ByoMap. Mean RT and RSD were calculated across the quadruplicate injections for each detected peak. The percent difference between means (DBM) was calculated between the mean RT for each RM 8671 peak and the mean RT for each corresponding PS 8670 peak. ‡two distinct peak shapes manually determined to share the same apex.

PS 8670		RM 8671		
**Mean TIC RT (min)**	**RSD (%)**	**Mean TIC RT (min)**	**RSD (%)**	**% Difference between means**
1.55	0.65	1.56	1.55	0.88
1.87	0.66	1.87	0.85	0.28
2.32	0.34	2.34	0.89	0.65
2.73	0.54	2.76	0.75	1.03
2.79	0.51	2.81	0.59	0.98
3.34	0.27	3.35	0.7	0.44
4.19	0.26	4.22	0.63	0.72
5.61	0.63	5.66	0.65	0.98
5.94	0.86	6.01	0.8	1.13
7.98	0.61	8.04	0.54	0.72
10.14	0.78	10.23	0.49	0.88
10.58	0.78	10.67	0.53	0.86
12.92	0.75	13.02	0.59	0.75
21.01	0.15	21.06	0.24	0.20
23.81	0.06	23.83	0.15	0.10
28.75	0.04	28.75	0.11	0.01
30.07	0.05	30.08	0.08	0.04
30.53	0.08	30.51	0.09	0.05
32.71	0.07	32.73	0.03	0.04
32.84	0.03	32.86	0.05	0.05
33.4	0.04	33.41	0.04	0.03
35.2	0.04	35.22	0.02	0.04
35.77	0.05	35.78	0.03	0.01
37.45	0.04	37.46	0.03	0.02
39.52	0.03	39.53	0.04	0.02
40.26‡ (a)	0.03	40.28‡ (a)	0.01	0.05
40.26‡ (b)	0.03	40.28‡ (b)	0.01	0.05
42.38	0.02	42.39	0.02	0.04
44.77	0.02	44.78	0.01	0.02

PS 8670			RM 8671		
Mean TIC RT (min)	RSD (%)		Mean TIC RT (min)	RSD (%)	% Difference between means
46.1	0.02		46.11	0.02	0.02
46.98	0.01		47	0.01	0.04
47.3	0.01		47.32	0.02	0.03
48.72	0.03		48.74	0.02	0.03
49.47	0.02		49.47	0.02	0.01
52.4	0.02		52.4	0.02	0.01
54.55	0.03		54.54	0.03	0.01
56.18	0.01		56.18	0.01	0.00
58.66	0.10		58.71	0.12	0.08
59.96	0.03		59.97	0.01	0.02
60.96	0.02		60.96	0.02	0.01
62.58	0.03		62.57	0.04	0.02
63.54	0.03		63.54	0.02	0.00
65.42	0.01		65.42	0.02	0.01
66.52	0.06		66.53	0.03	0.01
67.91	0.02		67.91	0.03	0.00
70.32	0.02		70.31	0.03	0.01
70.61	0.02		70.61	0.02	0.00
72.1	0.02		72.11	0.05	0.01
74.82	0.01		74.82	0.03	0.01
80.14	0.02		80.14	0.02	0.00
81.87	0.02		81.87	0.01	0.00
83.54	0.03		83.55	0.01	0.00
86.51	0.03		86.51	0.03	0.01
88.26	0.04		88.21	0.08	0.06

Table S4 Comparison of UV retention times between the PS 8670 reference map and RM 8671 Peptides generated from tryptic digestion of RM 8671 were analyzed by UV-LC-MS. UV peak retention times (RT) were extracted using Xcalibur. Mean RT and RSD were calculated across triplicate injections for each detected peak. The percent difference between means (DBM) was calculated between the mean RT for each RM 8671 peak and the mean RT for each corresponding PS 8670 peak.

**RM8670**	
**Mean UV RT (min)**	**RSD (%)**
1.78	0.28
1.89	0.00
1.93	0.00
2.71	0.00
2.98	0.17
3.26	0.25
5.12	0.29
5.55	0.66
5.86	1.00
6.55	0.36
7.91	0.60
10.07	0.78
10.51	0.80
14.74	0.40
20.94	0.16
23.75	0.07
28.67	0.07
30.00	0.04
30.42	0.04
32.64	0.03
32.78	0.03
33.33	0.02
35.13	0.01
35.69	0.03
37.03	0.03
37.37	0.03
37.67	0.00
39.43	0.00
40.18	0.01
42.31	0.01
44.70	0.00

**RM8671**		
**Mean UV RT (min)**	**RSD (%)**	**% Difference between means**
1.77	0.00	0.42
1.89	0.00	0.00
1.93	0.00	0.00
2.71	0.00	0.00
2.98	0.00	0.08
3.26	0.18	0.10
5.13	0.19	0.24
5.58	0.47	0.54
5.91	0.64	0.84
6.57	0.18	0.33
7.95	0.48	0.46
10.12	0.40	0.55
10.57	0.47	0.63
14.80	0.32	0.37
20.98	0.19	0.20
23.76	0.05	0.05
28.67	0.03	0.01
29.99	0.03	0.03
30.41	0.03	0.03
32.63	0.05	0.03
32.77	0.02	0.01
33.32	0.02	0.02
35.13	0.03	0.01
35.68	0.04	0.01
37.02	0.02	0.01
37.36	0.06	0.02
37.66	0.04	0.04
39.42	0.04	0.02
40.17	0.04	0.02
42.29	0.05	0.03
44.69	0.04	0.02

RM8670	
Mean UV RT (min)	RSD (%)
46.02	0.01
46.92	0.02
47.24	0.01
48.66	0.02
49.40	0.03
52.33	0.01
54.48	0.01
56.12	0.02
60.87	0.02
62.48	0.05
63.46	0.01
65.35	0.01
66.43	0.05
67.84	0.01
69.29	0.05
70.25	0.03
70.52	0.01
72.03	0.01
74.74	0.01
80.06	0.02
81.78	0.01
83.45	0.02
86.42	0.01
86.93	0.04
88.18	0.04

RM8671		
Mean UV RT (min)	RSD (%)	% Difference between means
46.01	0.04	0.03
46.90	0.04	0.03
47.23	0.03	0.02
48.64	0.04	0.03
49.39	0.04	0.02
52.31	0.04	0.04
54.46	0.06	0.03
56.10	0.05	0.03
60.85	0.05	0.03
62.46	0.06	0.03
63.44	0.05	0.04
65.32	0.05	0.05
66.33	0.09	0.15
67.81	0.05	0.04
69.25	0.04	0.06
70.22	0.05	0.04
70.50	0.05	0.03
72.00	0.04	0.04
74.71	0.05	0.04
80.04	0.04	0.03
81.75	0.03	0.03
83.42	0.04	0.04
86.40	0.04	0.03
86.90	0.05	0.03
88.16	0.07	0.02
